# Genomic Insights Into Energy Metabolism of *Carboxydocella thermautotrophica* Coupling Hydrogenogenic CO Oxidation With the Reduction of Fe(III) Minerals

**DOI:** 10.3389/fmicb.2018.01759

**Published:** 2018-08-03

**Authors:** Stepan V. Toshchakov, Alexander V. Lebedinsky, Tatyana G. Sokolova, Daria G. Zavarzina, Alexei A. Korzhenkov, Alina V. Teplyuk, Natalia I. Chistyakova, Vyacheslav S. Rusakov, Elizaveta A. Bonch-Osmolovskaya, Ilya V. Kublanov, Sergey N. Gavrilov

**Affiliations:** ^1^Laboratory of Microbial Genomics, Immanuel Kant Baltic Federal University, Kaliningrad, Russia; ^2^Winogradsky Institute of Microbiology, FRC Biotechnology, Russian Academy of Sciences, Moscow, Russia; ^3^Faculty of Physics, Lomonosov Moscow State University, Moscow, Russia

**Keywords:** *Carboxydocella*, thermophile, Kamchatka hot springs, genomics, hydrogenogenic carboxydotrophy, Fe(III) reduction, Fe(III) silicate minerals, Firmicutes

## Abstract

The genus *Carboxydocella* forms a deeply branching family in the class *Clostridia* and is currently represented by three physiologically diverse species of thermophilic prokaryotes. The type strain of the type species, *Carboxydocella thermautotrophica* 41^T^, is an obligate chemolithoautotroph growing exclusively by hydrogenogenic CO oxidation. Another strain, isolated from a hot spring at Uzon caldera, Kamchatka in the course of this work, is capable of coupling carboxydotrophy and dissimilatory reduction of Fe(III) from oxic and phyllosilicate minerals. The processes of carboxydotrophy and Fe(III) reduction appeared to be interdependent in this strain. The genomes of both isolates were sequenced, assembled into single chromosome sequences (for strain 41^T^ a plasmid sequence was also assembled) and analyzed. Genome analysis revealed that each of the two strains possessed six genes encoding diverse Ni,Fe-containing CO dehydrogenases (maximum reported in complete prokaryotic genomes), indicating crucial role of carbon monoxide in *C. thermautotrophica* metabolism. Both strains possessed a set of 30 multiheme *c*-type cytochromes, but only the newly isolated Fe-reducing strain 019 had one extra gene of a 17-heme cytochrome, which is proposed to represent a novel determinant of dissimilatory iron reduction in prokaryotes. Mössbauer studies revealed that strain 019 induced reductive transformation of the abundant ferric/ferrous-mica mineral glauconite to siderite during carboxydotrophic growth. Reconstruction of the *C. thermautotrophica* strains energy metabolism is the first comprehensive genome analysis of a representative of the deep phylogenetic branch Clostridia Incertae Sedis, family V. Our data provide insights into energy metabolism of *C. thermautotrophica* with an emphasis on its ecological implications.

## Introduction

Carbon monoxide (CO) is a common although minor component of gasses in terrestrial hot springs. In the hot springs of Yellowstone National Park (United States) and Uzon Caldera (Kamchatka, Russia), its concentration was reported to be about 1–6 10^-7^ mole fraction in the gas bubbling through the water (Shock et al., 2005, 2010) or 20–30 nM of dissolved gas (Kochetkova et al., 2011). The main sources of CO in hydrothermal vents are volcanic gasses (Menyailov and Nikitina, 1980; Symonds et al., 1994; Allard et al., 2004) and thermochemical or photochemical decomposition of organic matter (Hellebrand and Schade, 2008). Carbon monoxide is a strong reductant [*E^0′^*_(CO/CO2)_ is ca. -520 mV at pH 7.0], and can be involved in a number of microbial metabolic redox reactions (Shock et al., 2005, 2010; Diender et al., 2015) coupled with the reduction of various inorganic electron acceptors.

Key enzymes of CO oxidation pathways are carbon monoxide dehydrogenases (CODHs), which catalyze the oxidation of CO with water to CO_2_. CODHs from aerobes, encoded by *cox* genes, contain Cu and Mo as cofactors, while anaerobic CODHs, encoded by the distantly homologous *cooS* or *cdh* genes, contain Ni and Fe in active centers, where Ni activates CO and Fe provides the nucleophilic water. As distinct from the water-gas shift reaction, where the electrons and protons generated by CO oxidation are directly released as H_2_, CODHs keep the protons and electrons separated, and the electrons are transferred to the terminal acceptors via electron-carrier proteins. In case of hydrogenogenic carboxydotrophs and their [Ni,Fe]-CODHs, protons may serve as these terminal acceptors, and H_2_ is eventually released. [Ni,Fe]-CODHs are frequently associated with acetyl-CoA synthases (ACSs), where the [Ni,Fe]-CODH component reduces CO_2_ to CO, condensed with a methyl group and CoA by ACS to produce acetyl-CoA, or oxidizes CO formed upon acetyl-CoA cleavage (Jeoung et al., 2014).

A number of prokaryotes are capable of using CO as an electron donor, and some of them can also use CO as a carbon source (King and Weber, 2007; Oelgeschläger and Rother, 2008; Sokolova and Lebedinsky, 2013; Tiquia-Arashiro, 2014; Diender et al., 2015). In various terrestrial hydrotherms, potential activity or the presence of CO-oxidizing anaerobes has been revealed (Kochetkova et al., 2011; Brady et al., 2015; Yoneda et al., 2015), and the number of newly isolated CO-oxidizers is increasing permanently (Balk et al., 2009; Yoneda et al., 2012, 2013; Sokolova and Lebedinsky, 2013; Tiquia-Arashiro, 2014). Among cultivated thermophilic anaerobic CO-oxidizing species, hydrogenogenic carboxydotrophs are in majority, moreover, in certain hot springs, they comprise a significant portion of the microbial population (Yoneda et al., 2015). These bacteria oxidize carbon monoxide via the following reaction: CO + H_2_O → CO_2_ + H_2_ (ΔG^0′^ = -20 kJ). A representative of thermophilic hydrogenogenic CO-trophic microorganisms, *Carboxydocella thermautotrophica*, is the type species of the genus *Carboxydocella*, which forms a deep phylogenetic branch of the order Clostridiales, Incertae Sedis, family V (Sokolova et al., 2009; Sokolova, 2015). *Carboxydocella* species were found to be widely distributed in neutral hot springs with moderately thermophilic conditions (Slepova et al., 2006, 2007; Kochetkova et al., 2011; Slobodkina et al., 2012; Brady et al., 2015; Sokolova, 2015). So far, three *Carboxydocella* species have been validly described: *C. thermautotrophica* (Sokolova et al., 2002), *C. sporoproducens* (Slepova et al., 2006), and *C. manganica* (Slobodkina et al., 2012). While *C. thermautotrophica* and *C. sporoproducens* are carboxydotrophs, *C. manganica* is unable to oxidize CO.

Some thermophilic anaerobes isolated from various sedimentary environments of volcanic origin are capable of both hydrogenogenic CO-oxidation and dissimilatory ferric iron reduction from Fe(III) oxides (Sokolova et al., 2004; Slobodkin et al., 2006; Zavarzina et al., 2007; Slepova et al., 2009; Yoneda et al., 2012, 2013). At the moment, the interconnections between the oxidative and reductive branches of energy metabolism in these organisms remain unclear. Fe-bearing silicates (clay and mica minerals) comprising the most abundant Fe(III) source in volcanic areas (Eroschev-Shak et al., 1998, 2005) have not been tested as electron acceptors for growth of carboxydotrophs. Microbial redox transformation of these minerals is poorly understood so far, especially at elevated temperatures. Dissimilatory reduction of structural Fe from clays has been documented for a few thermophilic species (Pentráková et al., 2013). Less is known about bioreduction of Fe-rich micas, which are widely distributed in igneous and sedimentary rocks. The ability to reduce structural Fe in the mica mineral biotite was only demonstrated for resting cell suspensions of two mesophilic Fe(III)-reducing bacteria *Geobacter sulfurreducens* and *Shewanella oneidensis* (Brookshaw et al., 2014a,b). Bacteria of these species are the main models used for the investigation of microbial redox interactions with Fe(III) minerals, and the current concept of extracellular electron transfer in prokaryotes is mostly based on the data obtained for these microorganisms. Recent reviews of this concept (Shi et al., 2016; White et al., 2016) highlight the key role of multiheme *c*-type cytochromes in this process and the existence of two major pathways for direct electron transfer from respiratory chain to electron acceptor outside the cell. The first pathway is mediated by porin-cytochrome complexes linking intracellular electron shuttles and Fe(III)-reducing cytochromes on the cell surface. The second pathway is based on electrically conductive cell appendages (pili or “nanowires”). Genomic studies revealed that the determinants of both pathways are widespread among prokaryotes, although *in vivo* activity of porin-cytochrome complexes or nanowires has been documented for a restricted number of Fe(III)-reducers (Shi et al., 2012, 2014, 2016).

Here we report physiological and genomic characterization of two strains of *Carboxydocella thermautotrophica*. The type strain *C. thermautotrophica* 41^T^ is an obligate chemolithoautotroph growing exclusively by hydrogenogenic CO oxidation (Sokolova et al., 2002), while a new isolate of the same species, obtained during the current work (strain 019), differs significantly in its physiology, namely, by its capacity for Fe(III) reduction from oxic and mica minerals, which depends on CO availability. This physiological versatility of *C. thermautotrophica* strains has lead us to the proposal of an important ecological role for this taxon, such as coupling the transformation of carbon oxides and Fe minerals in terrestrial sedimentary environments of Kamchatka hot springs.

## Materials and Methods

### Strains

The type strain *C. thermautotrophica* 41^T^ (Sokolova et al., 2002) was obtained from DSMZ collection (DSMZ 1236). The strain 019 has been isolated from a core sample of the ground taken at East Thermal Field at Uzon Caldera (Kamchatka Peninsula, Russia).

### Sampling

A core was taken at East Thermal Field at Uzon Caldera (Kamchatka Peninsula) at an edge of Zavarzin Pool. Zavarzin Pool, 2.5 m in diameter, is formed by several hot springs discharging inside it. In the pool, thin brown layers of microbial mats were developed covered with a thick layer of sulfur. Temperature and pH of the water varied in different spots at the pool from 46 to 57°C and from 6.10 to 6.75, respectively. The core was taken at a 0.5-m distance from the pool and to 40-cm depth. The upper 10 cm of the core were black, next 15 cm in the middle had vertical black and white stripes, and the bottom 15 cm were almost white. The pH of the water filling the core was 5.8, its temperature was 55°C, and *E*_h_ was -225 mV (hereafter all the *E*_h_ values are presented vs. the potential of standard hydrogen electrode [SHE] at pH 6.5). Subsamples of top, middle and bottom layers of the core were taken anaerobically in tightly sealed bottles and designated as DC03-018, DC03-019, and DC03-020, respectively.

### Enrichment and Isolation

For enrichment and isolation of hydrogenogenic carboxydotrophic prokaryotes and their subsequent cultivation Media 1 and 2 were used. Medium 1 contained (g l^-1^): NH_4_Cl, 1; MgCl_2_^.^6H_2_O, 0.33; CaCl_2_^.^6H_2_O, 0.1; KCl, 0.33; KH_2_PO_4,_ 0.5; resazurin, 0.001; 1 ml l^-1^ of trace mineral solution (Kevbrin and Zavarzin, 1992); 1 ml l^-1^ of vitamin solution (Wolin et al., 1963), NaHCO_3_ (0.5 g l^-1^). Medium 1 was reduced with Na_2_S^.^ 9H_2_O (1.0 g l^-1^), and pH was adjusted to 6.8 with 6N HCl. Medium 2 had the composition similar to Medium 1 except the presence of hydromorphic ferric oxide (ferrihydrite), while sodium sulfide was omitted. Medium 1 had a redox potential characteristic of strictly anaerobic conditions (*E*_h_ value of -430 mV), and Medium 2 had a redox potential of -90 mV. Hereafter, strictly anaerobic conditions of Medium 1 are referred to as “low *E*_h_” and anaerobic conditions of Medium 2 are referred to as “high *E*_h_”. Microaerobic or aerobic growth conditions are specified where necessary. Yeast extract (0.5 g l^-^1) or sodium acetate (0.2 g l^-1^) or lactate (0.3 g l^-1^) were added as additional carbon sources when indicated. Glass vessels (60 ml total volume) sealed with butyl rubber stoppers (Bellco Glass Inc.) and containing 10 ml of liquid Medium 2 and 50 ml gas phase (100% CO) were inoculated with suspended core samples and incubated at 60°C. In chemical controls with non-inoculated media 1 or 2, equilibrium concentration of gaseous CO_2_, released due to thermal decomposition of bicarbonate at 60°C, was under the detection level at the beginning of incubation and did not exceed 0.03% (v/v) of the gas phase within further 6 days of incubation. Growth was determined using light microscopy and by monitoring CO utilization and gaseous products formation as described previously (Sokolova et al., 2002). After a number of serial end point dilution transfers in Medium 2 pure cultures were isolated from colonies obtained in roll-tubes prepared in 15-ml Hungate tubes with Medium 1 solidified by 5% agar, under 100% CO in the gas phase. Well-separated colonies were transferred to the liquid Medium 1.

### Physiological Studies

Utilization of molecular hydrogen, CO_2_, acetate, lactate (2 g/l) or glycerol (0.2%, v/v) by the new isolate was tested in liquid Medium 1 with 100% N_2_ or 80:20% H_2_/CO_2_ as the gas phase in presence or absence of potential electron acceptors. The acceptors tested were sodium nitrate, nitrite, sulfate, thiosulfate, AQDS, as well as mineral acceptors ferrihydrite (prepared as described by Gavrilov et al., 2012), glauconite (from Severo-Stavropol’skoye underground gas storage reservoir, Russia), nontronite (from the collection of clay minerals of the Faculty of Soil Science, Lomonosov Moscow State University) and diatomite (from Inzenskoye deposit, Russia). Soluble acceptors were added from pre-sterilized stock solutions to a concentration of 10 mM (5 mM in case of nitrite); Fe(III) minerals were added to Medium 2 before sterilization to achieve initial Fe(III) content of 90 mM in case of ferrihydrite and 20 mM in case of glauconite, nontronite, or diatomite.

CO, CO_2_, and H_2_ were analyzed using a ‘3700’ custom-modified gas chromatograph (ZIOC RAS Special Design Tech. Dept., Russia) supplied with Phoenix v.3.6.0 analytical software (BSoft, Russia) and equipped with zeolite NaX 80–100 mesh and Chromosorb-102 60-80 mesh 3 m columns and TCD detector, which were all conditioned at 30°C. Extra pure grade Ar was used as the carrier gas. Volatile fatty acids were determined by high performance liquid chromatography (HPLC) on a Stayer chromatograph (Aquilon, Russia) equipped with an Aminex HPX_87H column (Bio-Rad) and a Smartline 2300 refractometric detector (Knauer, Germany), with 5 mM H_2_SO_4_ as the eluent. Growth on iron-containing minerals was traced by the increase of the content of HCl-extractable Fe(II) in the mineral phase of Medium 2. The cell density was determined by direct cell counting under a CX41 phase-contrast microscope (Olympus). Temperature and pH growth optima were deduced from growth rates determined on Medium 1.

### Mössbauer Studies

Mössbauer spectra of ^57^Fe nuclei were recorded at room temperature on a custom-modified MS-1104Em spectrometer (Research Institute of Physics, Southern Federal University, Russia), which was equipped with a ^57^Co-source in a Rh matrix and operated in a constant acceleration mode. The spectrometer was calibrated using standard α-Fe absorbent. The SpectrRelax software (Matsnev and Rusakov, 2014) was used to analyze the Mössbauer spectra. Iron species were determined colorimetrically in HCl extracts of culture subsamples with ferrozine (Fe^2+^) or potassium thiocyanate (Fe^3+^), as previously described (Gavrilov et al., 2012). Insoluble parts of silicate minerals were separated from HCl extracts by centrifugation at 12 kG for 10 min on an Eppendorf table top centrifuge. Non-extractable iron content of glauconite was estimated from Mössbauer spectral data under the assumption that the recoil-free fraction (probability of Mössbauer effect) is equal for iron atoms located in different positions in various mineral phases, and accordingly, relative intensities of subspectra (area of subspectra) are equal to the relative content of iron atoms in these positions.

### DNA Isolation

For DNA isolation cells were cultivated on Medium 1 in the absence of minerals under optimal conditions, collected by centrifugation at 9 kG for 15 min and disrupted with glass beads using Minilys homogenizer (Bertin Technologies). DNA was extracted using QIAamp DNA mini kit (Qiagen).

### Phylogenetic Reconstructions

Phylogenetic trees were constructed using the Maximum Likelihood method based on the Jones–Taylor–Thornton model (Jones et al., 1992) in MEGA6 (Tamura et al., 2013).

For the putative iron reducing cytochrome, separate reconstructions were performed for C-terminus (first 500 aa residues) and the remaining part of the sequence (501–1486 aa residues), containing all the conservative multiheme cytochrome domains (i.e., putative “catalytic” domain of the protein). Blastp analysis was performed against UniProtKB prokaryotic database on April, 2018. For the catalytic domain of the protein, only the hits with *E*_v_ < 0.001, query coverage ≥50% and identity >20% (which is relevant for multiheme cytochromes, Sharma et al., 2010) from cultivated strains were considered. The analysis retrieved 57 sequences, which were then filtered through 0.9 filter using CD-hit utility^1^ and the resulting set of 50 sequences was aligned with built-in Muscle at default parameters in MEGA6.

### Genome Sequencing and Assembly

Sequencing projects were started in May 2013 and finished in August 2014. For sequencing of the genomes of *C. thermautotrophica* strains both paired-end and mate-paired DNA libraries were used. Paired end libraries were prepared from 1 μg of genomic DNA with NEBNext^TM^ Ultra DNA library preparation kit (New England Biolabs, United States) according to manufacturer’s instructions to obtain mean library size of 500 bp. Mate-paired libraries were prepared with Nextera^TM^ Mate Pair Library Prep Kit (Illumina Inc., United States) using bead-based size selection protocol. Both paired-end mate-paired libraries were sequenced using 2 × 250 bp reads with MiSeq^TM^ Personal Sequencing System (Illumina Inc., United States). After sequencing all reads were subjected to stringent quality filtering with CLC Genomics Workbench 8.5 (Qiagen, Germany). After filtering, overlapping paired-end library reads were merged with SeqPrep tool^2^, resulting in 215,048 and 227,940 single merged reads; and 895,488 and 892,408 read pairs for strains 41^T^ and 019, respectively. Fragment size of both paired-end libraries was 500–700 bp. Mate-paired reads were treated with NextClip tool (Leggett et al., 2014), resulting in 2,382,016 and 2,123,106 read pairs with mean insert sizes of 2,439 and 2,382 bp for strains 41^T^ and 019, respectively. Reads were assembled with both ALLPATHS-LG (Butler et al., 2008) and SPADES 3.8.0 (Nurk et al., 2013) assemblers and refined manually using CLC Genomics Workbench 8.5 software (Qiagen, Germany). Orientation of contigs and final filling of sequence gaps was made by PCR with outward-oriented primers to contigs termini and subsequent Sanger sequencing of resulting amplicons. Final average genome coverage was 247× and 218× for strains 41^T^ and 019, respectively. During assembly of strain 41^T^ genome an additional circular contig, corresponding to plasmid, was also assembled.

The complete genome sequences of the *C. thermautotrophica* type strain 41^T^ and strain 019 have been deposited in DDBJ/EMBL/GenBank under accession numbers CP028491 (strain 019), CP028514 (strain 41^T^) and CP028515 (plasmid of strain 41^T^). Related projects information and sample details have been deposited in NCBI database under accession numbers PRJEB11520, PRJEB11521, and SAMN07757920, SAMN07757921, respectively.

### Genome Annotation

Gene prediction and primary annotation was performed with IMG/M ER System (Chen et al., 2017). Refining of the automated annotations and other predictions were done manually according to genome annotation protocol (Toshchakov et al., 2015).

Subcellular localization of multiheme cytochromes was predicted based on the results of six different on-line prediction services – SignalP 4.1, TatP 1.0, SecretomeP 2.0a and TMHMM 2.0 (all at CBS Prediction Servers^3^), as well as PSORTb 3.0.2^4^ and Phobius^5^.

Conserved domains were predicted with HMMSCAN^6^ considering Pfam, TIGRFAM, Gene3D, Superfamily, PIRSF and TreeFam protein families databases.

## Results

### Enrichment and Isolation of Strain 019, Cell Morphology of the New Isolate

The *C. thermautotrophica* Fe(III)-reducing strain 019 was obtained from a core sample taken near Zavarzin thermal pool at Uzon Caldera (Kamchatka). The bottles with anaerobic Medium 2 (high *E*_h_) containing sodium acetate and ferrihydrite under a 100% CO atmosphere and inoculated with the middle (black-to-white) part of the core sample (subsample DC03-019) showed an increase in pressure from 140 to 170 kPa after 2 days of incubation at 60°C. Growth of small rod-shaped cells was observed. The CO content in the gas phase decreased to 40% and about 30% H_2_ and 30% CO_2_ were formed. After several transfers in the same medium the enrichments were serially tenfold diluted and transferred to the Medium 1 (low *E*_h_) with sodium acetate and without Fe(III) under 100% CO, and then to roll-tubes with the same solidified Medium 1. After 2 days of incubation at 60°C, round white colonies about 0.5 mm in diameter were observed, which were transferred to a similar liquid medium. As a result, an isolate designated strain 019 was obtained.

Cells of the isolate 019 were straight rods with a length of 1–1.5 μm and a width of about 0.4–0.5 μm, arranged singly or in pairs. Cells were motile due to lateral flagella but singular cell aggregates on magnetite were also observed (Supplementary Figure S1).

### Growth Characteristics of the New Isolate

#### Physico-Chemical Parameters

Growth of strain 019 was observed in the temperature range of 45–68°C, with an optimum at 58°C, and in the pH range 6.5–7.6, with an optimum at pH 7.0. Strain 019 grew anaerobically in a wide range of culture conditions: autotrophically or heterotrophically, by respiration or by fermentation. Aerobic or microaerobic growth was not observed with any of the tested concentrations of oxygen in the gas phase, i.e., under 100% air, a mixture of CO and air (4:1 v/v), or CO with 0.5, 1, or 2% O_2_.

#### Carbon Substrates

Autotrophic growth of strain 019 under CO was observed in the absence of any organic carbon sources. H_2_ as the electron donor with Fe(III), thiosulfate or nitrate as electron acceptors did not support autotrophic growth under a H_2_/CO_2_ gas phase. Organic substrates either stimulated carboxydotrophic growth or supported the growth in the absence of CO. Acetate and lactate but not pyruvate were utilized concomitantly with complete CO oxidation and hydrogen formation, the presence of the organic acid salt raising the cell yield up to 10 times compared to autotrophic growth conditions. Fermentative growth of strain 019 was observed on yeast extract, sucrose, glucose, maltose, or pyruvate under pure N_2_ or CO atmosphere. Galactose, cellobiose, cellulose, lactose, glycerol, peptone were not utilized. Glucose was fermented to lactate, acetate and H_2_.

#### Electron Donors and Acceptors

**FIGURE 1:**
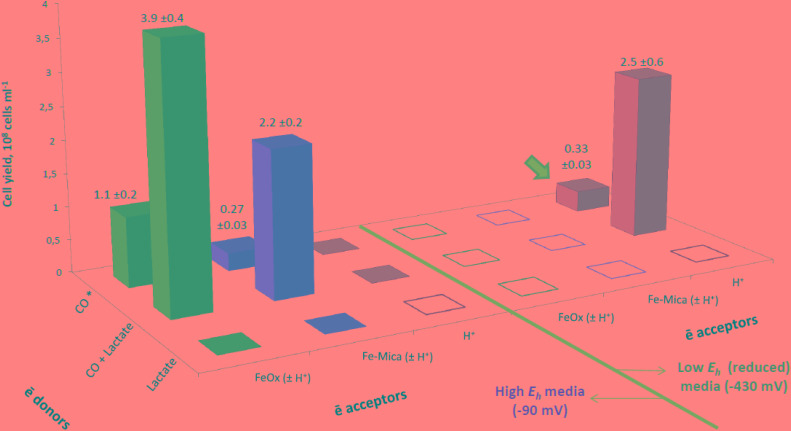
Cell yield of strain 019 cultures as dependent on availability of Fe(III), organic carbon and a reductant in the medium. Indicated above the bars are cell yields and corresponding error limits (×10^8^ cells ml^-1^). Note that two types of anaerobic culture media (high *E*_h_ and low *E*_h_) were used to test each couple of electron donor and acceptor with both *C. thermautotrophica* strains. The initial *E*_h_ values were identical in the culture media of a particular type and are indicated on the diagram. Low *E*_h_ medium was pre-reduced with sodium sulfide (refer to “Materials and Methods” section for details). Electron donor/acceptor couples which have been tested but did not support the growth of a *C. thermautotrophica* strain are shown as flat filled squares. Empty squares indicate donor/acceptor couples which are biochemically senseless (lactate and H^+^) or physico-chemically restricted [rapid chemical reduction of Fe(III) minerals by sulfides]. Asterisk indicates autotrophic growth conditions when CO was the only carbon source and electron donor available in the culture medium. Thick arrow indicates the conditions supporting the growth of the type strain 41^T^; data on its cell yields are not presented.

During autotrophic growth on CO coupled to hydrogenogenic carboxydotrophy, strain 019 utilizes protons as electron acceptors in its energy metabolism. Among electron acceptors other than protons, only Fe(III) stimulated growth of the strain, but it was exclusively utilized in the presence of CO (**Figure 1**). Thiosulfate and nitrate did not affect growth of the organism with organic acids under CO and inhibited autotrophic growth under CO (data not presented). Sulfate, nitrite or AQDS inhibited growth of strain 019 under any of the conditions tested.

Utilization of protons and Fe(III) as electron acceptors by strain 019 during autotrophic or heterotrophic (with lactate) growth appeared to depend on redox potential of the culture medium (**Figure 1**). In high *E*_h_ anaerobic medium, the growth was only possible in the presence of both CO and an Fe(III) mineral. In contrast, in low *E*_h_ culture medium, strain 019 grew either by carboxydotrophy in the absence of electron acceptors other than protons or by fermentation, i.e., with organic electron donors in the absence of both CO and Fe(III). Checking the Fe(III) reducing ability of strain 019 in low *E*_h_ medium appeared to be impossible due to rapid chemical redox interactions at 60°C between insoluble Fe(III) and sulfides, added to decrease the redox potential.

In parallel experiments, the type strain 41^T^ grew only at low *E*_h_ by autotrophic hydrogenogenic CO oxidation without additional electron acceptors (**Figure 1**), as described previously by Sokolova et al., 2002.

### Fe(III) Reduction From Minerals by Strain 019

The growth of strain 019 in the presence of both Fe(III) and CO was accompanied by Fe(III) reduction, complete CO oxidation and the formation of apparently equimolar quantities of CO_2_ and H_2_. In our experiments we used insoluble Fe(III) forms, which are typical for physico-chemical conditions in natural habitats of *C. thermautotrophica*. No soluble Fe(II) or Fe(III) was detected in cultures during their growth with various Fe(III) minerals.

#### Fe(III) Reduction From Ferrihydrite

**FIGURE 2:**
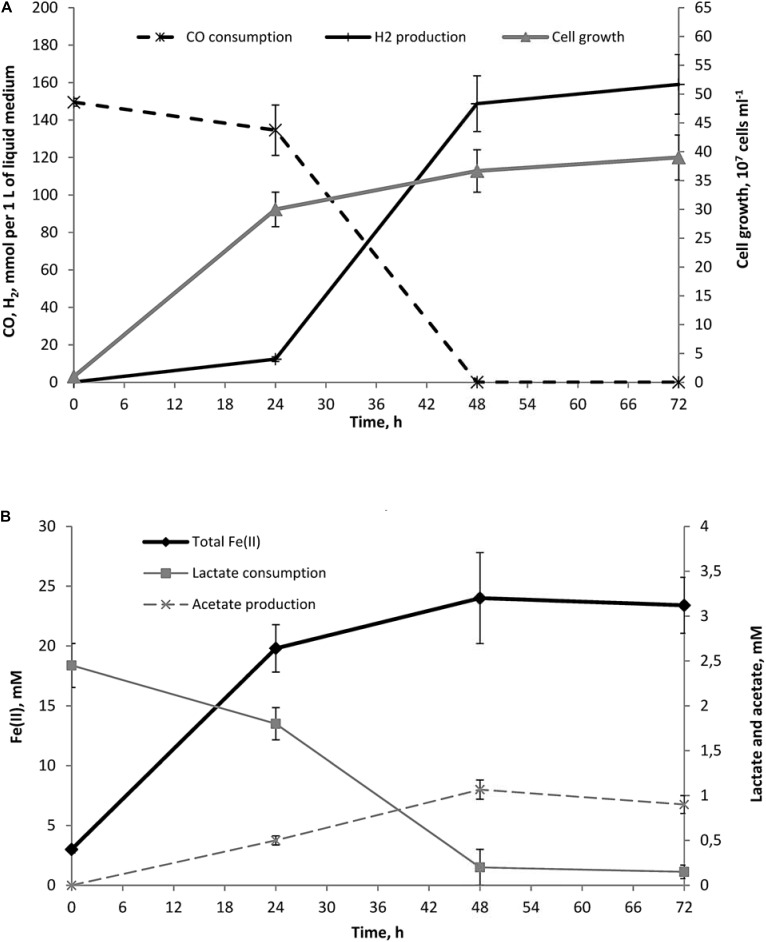
Dynamics of growth, Fe(II) production, lactate consumption, acetate formation and concomitant hydrogenogenic carboxydotrophy of *C. thermautotrophica* strain 019 growing on CO and lactate with ferrihydrite. **(A)** Cell growth, CO consumption and H_2_ production; **(B)** Fe(II) production, lactate consumption and acetate formation.

Fe(III) supplied as an oxic mineral ferrihydrite provided maximal cell yield of strain 019 (**Figure 1**). During the growth, about 23% of insoluble Fe(III) from ferrihydrite was reduced to Fe(II) in the form of a black magnetic mineral (presumably, magnetite) with concomitant decrease of the culture *E*_h_ down to -360 mV. The cell yield of autotrophic cultures grown on CO increased three times in the presence of ferrihydrite, in spite of the high initial *E*_h_ of the medium. Lactate stimulated the growth on CO with ferrihydrite (**Figure 1**). Consumption of 2.3 mM lactate was accompanied by the formation of 1.1 mM acetate (**Figure 2B**), indicating that about half of the oxidized lactate was utilized as an electron donor in catabolic reactions, while the rest of lactate was likely consumed as a carbon source. In contrast, only 0.6 mM lactate was consumed by cultures grown on CO and lactate in the absence of Fe(III) at low *E*_h_, and no acetate production was detected in these growth conditions. Kinetic experiments revealed that maximal growth rate in high *E*_h_ medium containing ferrihydrite, CO and lactate was observed within the first 24 h of incubation simultaneously with the highest rates of Fe(III) reduction and lactate conversion to acetate. About 77% of the lactate consumed by the end of this growth phase was converted to acetate (**Figure 2B**), while only 10% of CO was oxidized with equimolar H_2_ formation. Maximal rate of hydrogenogenic carboxydotrophy was achieved later, when cell growth and Fe(III) reduction slowed down (**Figure 2**).

#### Fe(III) Reduction From Phyllosilicates

**FIGURE 3:**
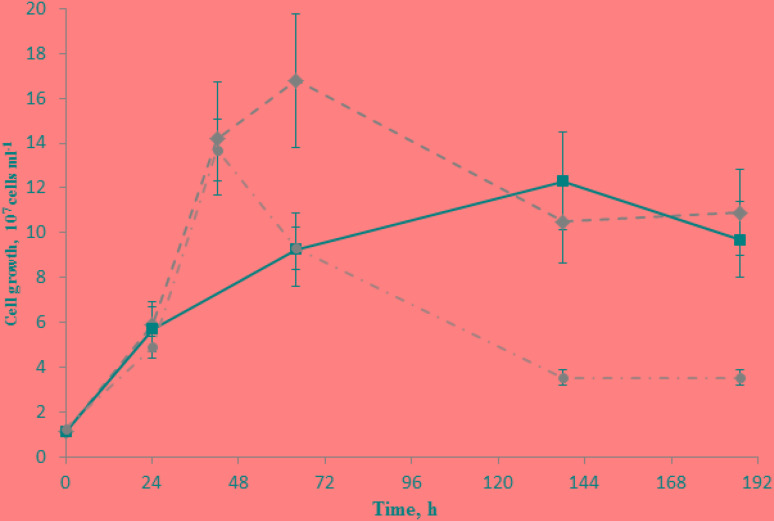
Growth of *C. thermautotrophica* strain 019 with Fe(III)-containing silica mineral and rock on lactate under 100% CO. Diamonds – growth with glauconite at high *E*_h_ (–90 mV); squares – growth with diatomite at high *E*_h_; circles – control growth without external electron acceptors at low *E*_h_ (–430 mV).

**FIGURE 4:**
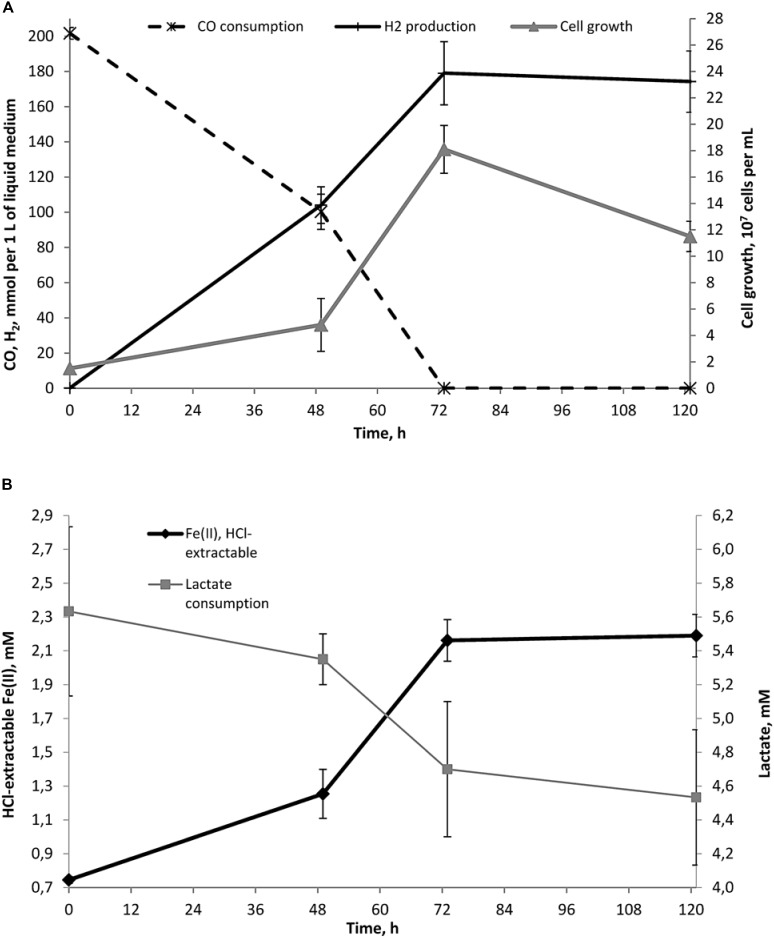
Dynamics of growth, Fe(II) production, lactate consumption and concomitant hydrogenogenic carboxydotrophy of *C. thermautotrophica* strain 019 growing on CO and lactate with glauconite. **(A)** Cell growth, CO consumption and H_2_ production; **(B)** lactate consumption and Fe(II) formation. No acetate production has been recorded during the growth with glauconite.

**FIGURE 5:**
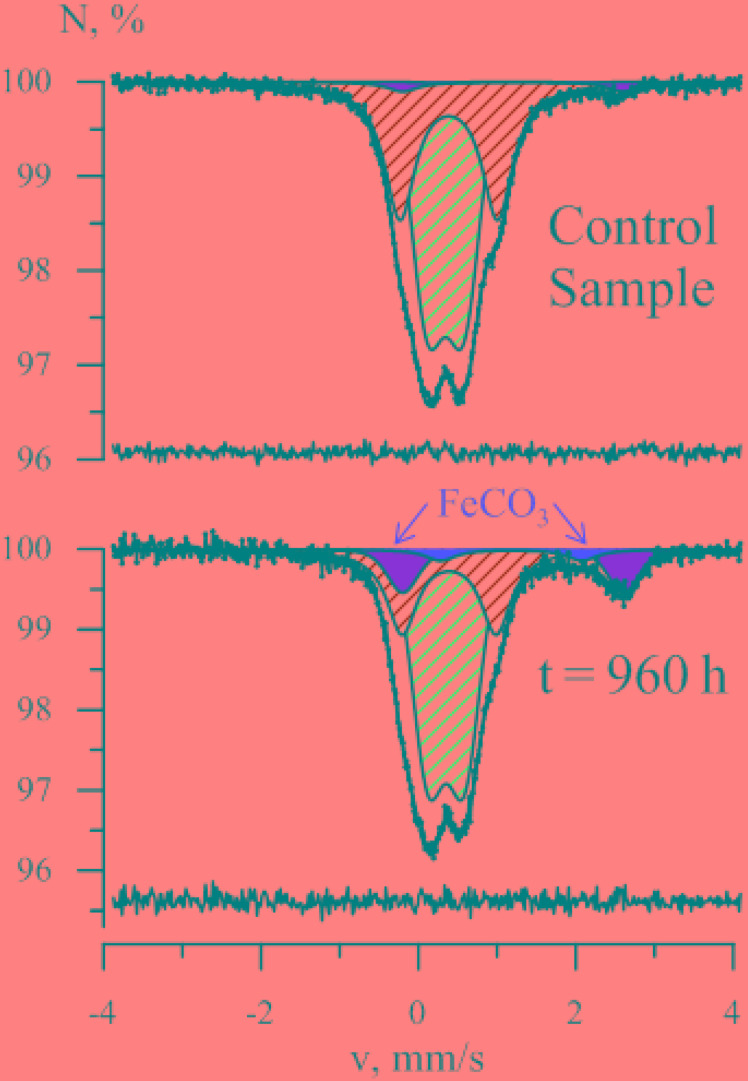
Mössbauer spectra of the mineral phase formed in glauconite-reducing culture of *C. thermautotrophica* strain 019 grown with CO and lactate. Characteristic peaks of siderite are marked red. Samples of the mineral phase were collected for analysis at the 960th hour of incubation after complete cell lysis.

As strain 019 was isolated from a mineral core sample, it was tested for the ability to utilize structural Fe(III) from crystal lattice of phyllosilicates – the group of minerals previously detected in Zavarzin hot pool (Rozanov et al., 2014) right nearby the site where samples for our enrichments were obtained. We tested mixed valence Fe(III/II) minerals: the Fe-mica glauconite, clay mineral nontronite and siliceous sedimentary rock diatomite containing admixtures of natural Fe oxides (Distanov, 1987). Growth of strain 019 was observed in three consecutive transfers with diatomite and glauconite (**Figure 3**). Growth with diatomite was observed only in the presence of lactate as a potential carbon source, while growth with glauconite was observed both in the presence and in the absence of organic substrates. Growth with glauconite correlated with an increase in HCl-extractable Fe(II) (**Figure 4**) and non-extractable structural Fe(II) content (Supplementary Figure S2) of the Fe-mica mineral. During autotrophic growth, only a minor increase of glauconite Fe(II) content (by ca. 0.2 mM) was observed and the cell yield correlated with that detected in the absence of Fe(III) minerals at low *E*_h_. Lactate stimulated the growth with glauconite 10-fold (**Figure 1**) and Fe(II) production from glauconite sevenfold (up to 1.42 mM). About 1 mM of lactate was consumed concomitantly with glauconite reduction but no acetate production was detected, indicating utilization of lactate as the carbon source only (**Figure 4B**). The growth was accompanied by a pronounced decrease of *E*_h_ down to -520 mV. In a control experiment, simulating the initial Fe(III)/Fe(II) ratio of glauconite by using a mixture of ferrihydrite and magnetite, the *E*_h_ value of the cultures rapidly decreased from -90 to -320 mV within the first 8 h of incubation. Changes in *E*_h_ of uninoculated controls with glauconite or ferrihydrite/magnetite mixture were much lower. The growth of strain 019 in the presence of lactate, CO and glauconite started with active CO oxidation and H_2_ formation. The rates of lactate consumption and Fe(III) reduction were minimal in this growth phase, but after 49 h of incubation, maximal rates of growth and of all of the mentioned metabolic processes were achieved simultaneously (**Figure 4**). Fe(III) reduction from the mica mineral by strain 019 rapidly ceased upon exhaustion of CO, followed by a start of cell lysis (**Figure 4**). However, to trace possible minor structural changes in glauconite, induced by microbial Fe(III) reduction but not leading to an increase in the HCl-extractable Fe(II) content of the mineral, we continued incubation of the cultures further on. Mössbauer investigations revealed that the relative amounts of Fe^2+^ and Fe^3+^ atoms in glauconite remained virtually constant within the first 78 h of incubation, until cell lysis started. During subsequent incubation, the relative content of ferrous atoms in glauconite increased almost twice from 2.2 ± 0.5% to 5.7 ± 0.4% by the 166th hour, and this trend was strengthening within further 794 h of incubation, until all the cells have been completely lysed (Supplementary Figure S2). Mössbauer spectra of the studied samples within all the incubation period were of paramagnetic type with a superposition of quadrupole doublets. Final spectra, captured at the 960th hour (**Figure 5**), were clearly fitted by four quadrupole doublets with equal line widths, depicting the formation of a small relative amount of a new mineral phase containing Fe^2^^+^ atoms in the octahedral oxygen environment. This phase was identified as siderite (FeCO_3_), which indicated the reduction of Fe^3+^ atoms in glauconite lattice structure. The relative concentration of siderite in the sample, obtained at the end of the entire 960-h incubation period, was *I* = 3.9 ± 1.3%. No changes in the mineral structure were detected in abiotic controls (**Figure 5**), as well as no production of H_2_ or Fe(II), no decrease in lactate concentration, and no interactions of CO or H_2_ with Fe(III) were observed within the same incubation period.

No growth or Fe(III) reduction from any of the tested Fe(III) minerals was observed with the type strain 41^T^ under the same cultivation conditions. Neither could the type strain grow by fermentation.

### Phylogenetic Position of Strain 019

The average nucleotide identity (ANI) value between the genomes of strains 019 and 41^T^, calculated using ANI calculator^7^ with default parameters, was 99.7%; while the species-delimiting value, corresponding to the 70% level of *in vitro* DNA–DNA hybridization, is 95% (Goris et al., 2007). Thus, *in silico* hybridization of the genomes shows the affiliation of strains 019 and 41^T^ with the same species, *Carboxydocella thermautotrophica*.

### Genomic Properties of *C. thermautotrophica* Strains 019 and 41^T^

The genome of *C. thermautotrophica* type strain consists of one circular chromosome of a total length of 2690058 base pairs (49,14% GC content) and one circular plasmid of 53067 nucleotides (41% GC content). Read coverage of plasmid was 3.5 times higher than average chromosome coverage, suggesting 3–5 copies of plasmid per cell. The length of *C. thermautotrophica* strain 019 circular chromosome is 2676584 base pairs (49,14% GC content), and no plasmids have been identified in this strain.

**FIGURE 6:**
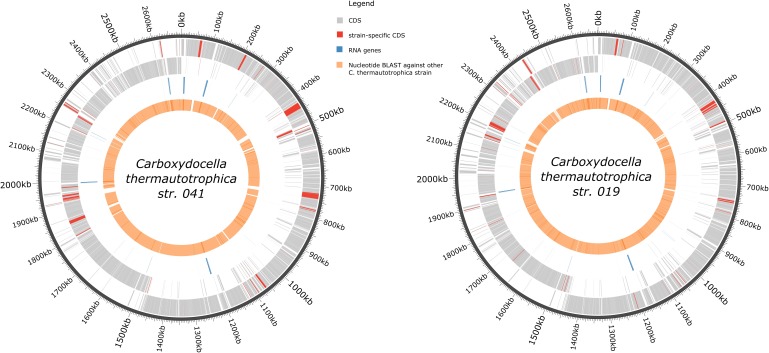
Graphic representation of the genomes of *C. thermautotrophica* strains. Rings from outside to inside: genomic coordinates (dark gray); plus-strand common (gray) and strain-specific (red) CDSs; minus-strand common (gray) and strain-specific (red) CDSs; RNA genes (blue); blastn hits with e-value cutoff 10^-5^ vs. the other *C. thermautotrophica* strain.

For the *C. thermautotrophica* 041^T^ chromosome, 2810 genes were predicted, 2697 of which are protein-coding genes and 113 are RNA genes (**Table 1**). 65.5% of genes were assigned to at least one COG cluster with IMG annotation pipeline (Huntemann et al., 2015). The distribution of hits to COG functional categories is presented in Supplementary Table S1. Genome has five complete ribosomal operons with 16S rRNA genes showing at least 99.6% identity with each other. General genomic features of *C. thermautotrophica* strain 019 were similar to those of the type strain chromosome and are presented in **Table 1** and **Figure 6**.

**Table 1:** General features of replicons of *C. thermautotrophica* strains.

	*C. thermautotrophica* 019 chromosome	*C. thermautotrophica* 041^T^ chromosome	*C. thermautotrophica* 041^T^ plasmid
Replicon length, bp	2676584	2690058	53067
GC mol %	49.14	49.14	41.3
Number of genes	2809	2810	51
RNA genes	120	113	0
tRNA	71	73	0
rRNA	15	15	0
Protein-coding genes	2689	2697	51
Assigned to COG	1775	1787	14
Number of GIs	9	8	NA
Total length of GIs, bp	214072	211619	NA


The plasmid of strain 41^T^ encoded 51 proteins, 21 of their ORFs showed dispersed weak to moderate (23–58%) hits to the chromosome-encoded *in silico* proteomes of both strains, and two of the ORFs revealed weak hits to the proteome of strain 019 only. Little if any of these proteins are of clear metabolic relevance. Presence in the plasmid of the IS1182 mobile element, along with genes of restriction-modification system, suggests that it may be a selfish mobile element (Kobayashi, 2001; Koonin, 2011).

### Comparative Genome Analysis of CO and Fe(III) Metabolism in *C. thermautotrophica* Strains 019 and 41^T^

#### Oxidative Phosphorylation

A set of genes of the proton-translocating type I NADH-dehydrogenase (complex I) *nuoABCDHIJKLMN* is encoded in the same order by CFE_1311–1321 in strain 019 and CTH_1331–1341 in strain 41^T^. Subunits NuoEFG, essential to provide the catalytic site for NADH oxidation, are encoded separately in both genomes: CFE_2217–2219 in strain 019 and CTH_2319–2321 in strain 41^T^. NuoEFG proteins in both strains share weak sequence similarity with their homologs from UniProt database. Gene clusters of the respiratory complex II (succinate dehydrogenase) are duplicated in both genomes (CFE_1726–1728 and CFE_2117–2119 in strain 019, and CTH_1740–1742 and CTH_2150–2152 in strain 41^T^). Oxidative phosphorylation in both strains is performed via F_0_F_1_-type bacterial ATP-synthases, encoded by CFE_0343–0352 in strain 019 and CTH_0352–0361 in strain 41^T^.

#### Inorganic Carbon Assimilation

Both *C. thermautotrophica* strains possess full sets of genes for the Wood–Ljungdahl pathway of inorganic carbon assimilation. The genes of the so-called Western (carbonyl) branch of the pathway and genes of the final steps of the Eastern (methyl) branch are encoded in each of the strains in a large gene cluster (Supplementary Table S2) similar to the *acs* gene cluster of *Moorella thermoacetica* ATCC 39073 (Pierce et al., 2008), a model organism to study the Wood–Ljungdahl pathway. Interestingly, in *C. thermautotrophica* strains additional genes of methylenetetrahydrofolate reductase subunits MetVF are also present in a smaller gene cluster (Supplementary Table S2), and here they occur together with *hdrCBA* and *mvhD* genes, which in *M. thermoacetica* adjoin the *metVF* genes in the large *acs* cluster. Other Wood–Ljungdahl pathway genes of *C. thermautotrophica* strains are dispersed over the chromosomes (Supplementary Table S2), as it is in *M. thermoacetica*. Genes encoding key enzymes of other known autotrophic pathways (Fuchs, 2011; Nunoura et al., 2018; Mall et al., 2018) could not be found.

Both *C. thermautotrophica* strains also possess genes that might extend the anabolic function of the Wood–Ljungdahl pathway to the catabolic capacity for acetogenesis, including acetogenesis on H_2_ + CO_2_. The genomes harbor genes of phosphotransacetylase (two non-homologous genes in each genome) and acetate kinase (Supplementary Table S2), which provide for acetate and ATP production from acetyl-CoA and ADP + P_i_, and genes encoding close homologs (49–73% amino acid sequence identity, Supplementary Table S2) of the subunits of enzymatic complexes that promote reductant balance and energy conservation during acetogenesis by *M. thermoacetica* (Schuchmann and Müller, 2014; Basen and Müller, 2017), including an energy converting hydrogenase additional to the CO-induced one, considered in the next subsection.

However, despite the apparent presence of all required acetogenesis determinants, we failed to obtain growth of strains 41^T^ and 019 on H_2_ + CO_2_ mixture or to detect acetate production during autotrophic growth under CO.

#### Genomic Determinants of Carboxydotrophy

A common metabolic feature of *C. thermautotrophica* strains is their ability to grow at the expense of hydrogenogenic oxidation of carbon monoxide. In the type strain 41^T^, this ability appeared to be restricted by the redox potential of the culture medium, manifesting itself only at its low values. In contrast, strain 019 grew by CO oxidation also at relatively high *E*_h_ of -90 mV, but only in the presence of Fe(III) minerals, which were utilized as electron acceptors in parallel with protons.

**FIGURE 7:**
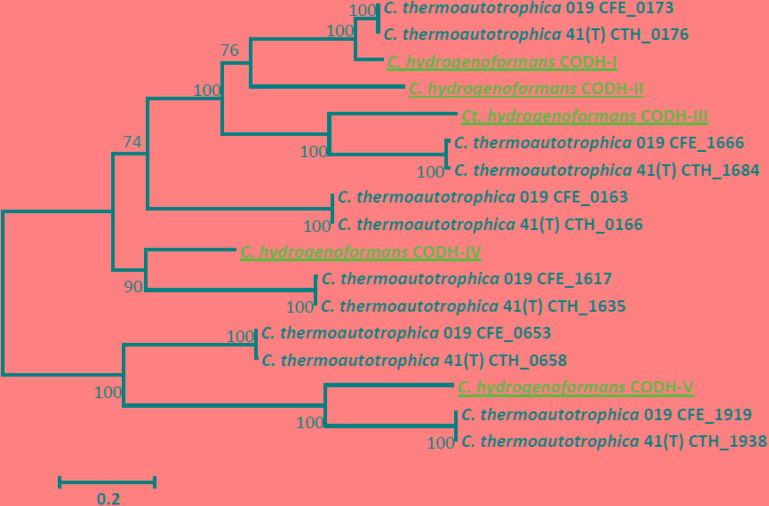
Phylogenetic tree of *Carboxydocella thermautotrophica* [Ni,Fe]-CODHs. The tree was constructed by Maximum Likelihood method using MEGA6 (Tamura et al., 2013) at default parameters after aligning sequences with built-in Muscle at default parameters. Underlined blue are the [Ni,Fe]-CODHs of *Carboxydothermus hydrogenoformans*, which were used as references for function prediction.

Each of the genomes of *C. thermautotrophica* strains 41^T^ and 019 harbors six *cooS* genes, encoding anaerobic (Ni,Fe-containing) CODHs, and no genes encoding aerobic (Mo,Cu-containing) CODHs. Quite recently (see the “Discussion” section for the latest data), the highest number of [Ni,Fe]-CODHs encoded in available completely sequenced genomes was five, one of such few genomes belonging to the well-studied hydrogenogenic carboxydotroph *Carboxydothermus hydrogenoformans* (see Svetlitchnyi et al., 2004; Wu et al., 2005 for substantiation of the integrity of the *cooS3* gene in the original isolate of *C. hydrogenoformans*). The functional roles of the five [Ni,Fe]-CODHs of *C. hydrogenoformans* have been studied by various approaches, and for four of them functions have been established (Svetlitchnyi et al., 2001, 2004; Soboh et al., 2002; Hedderich, 2004; Wu et al., 2005). The phylogenetic relations *C. thermautotrophica* [Ni,Fe]-CODHs with the [Ni,Fe]-CODHs of *C. hydrogenoformans* are shown in **Figure 7**.

**FIGURE 8:**
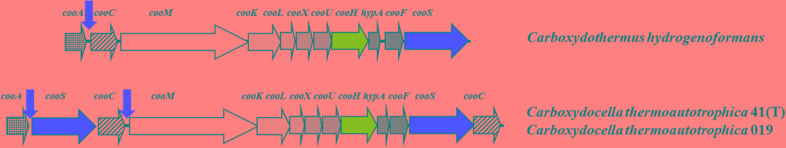
Gene clusters determining hydrogenogenic carboxydotrophy in *Carboxydothermus hydrogenoformans* and *Carboxydocella thermautotrophica* strains 41^T^ and 019. *cooA*: gene of redox- and CO-sensitive transcriptional regulator; *cooS* (red horizontal arrow): [Ni,Fe]-CODH gene; *cooC*: gene of [Ni,Fe]-CODH accessory nickel-insertion protein; *cooMKLXUH*: genes of energy-converting hydrogenase (ECH); *hypA*: gene of hydrogenase maturation protein; *cooF*: gene of ferredoxin-like protein; *cooH* (blue horizontal arrow): gene of ECH catalytic subunit. Red vertical arrows show locations of CooA-binding sites. The locus tags for the gene clusters are CHY_1835-1824, CTH_0165-0177, and CFE_0162-0174. Note that the exact role of the upstream ‘enigmatic’ *cooSC* operon (CTH_0166-167 and CFE_0163-0164) in carboxydocellas remains unclear (refer to the text for details).

The roles of two [Ni,Fe]-CODHs in each *C. thermautotrophica* strain can be deduced from high similarity of their amino acid sequences and genomic contexts with [Ni,Fe]-CODH-I and [Ni,Fe]-CODH-III of *C. hydrogenoformans*. The *cooSI*-like genes CFE_0173 and CTH_0176 are parts of gene clusters (**Figure 8**) highly similar to the *C. hydrogenoformans* gene cluster that includes, along with *cooSI*, genes of an energy-converting hydrogenase (ECH) and is responsible for hydrogen formation from CO + H_2_O with transmembrane potential generation (Svetlitchnyi et al., 2001, 2004; Soboh et al., 2002; Hedderich, 2004). However, in the *C. thermautotrophica* strains this gene cluster has a more complicated pattern, including a second *cooC* gene in the downstream region and a second *cooS* gene (CFE_0163 and CTH_0166) in the upstream region, between the *cooC* gene and the *cooA* gene of the CO- and redox-sensing transcription regulator protein CooA (Roberts et al., 2001). This additional *cooS* gene does not have close homologs in *C. hydrogenoformans.*

The operon formula of the cluster in *C. thermautotrophica* strains is apparently *cooA-cooSC-cooMKLXUHhypAcooFSC-*, i.e., the two [Ni,Fe]-CODHs are encoded in distinct operons. However, these operons are evidently co-regulated by the above-mentioned CO- and redox-sensing regulator protein CooA. In both *C. thermautotrophica* strains, CooA-specific binding sites, conforming to the long-known consensus formula TGTCRNNNNNNYGACR (Fox et al., 1996), are located upstream of the *cooSC* and *cooMKLXUHhypAcooFSC* operons (**Figure 8**). The role of the *cooSC* operon is obscure, but the fact that it is regulated by CooA, sensing CO at low redox potential conditions (Roberts et al., 2001), provides grounds to speculate that the [Ni,Fe]-CODHs CFE_0163 and CTH_0166 have a role to play in CO oxidation with hydrogen production.

The [Ni,Fe]-CODHs of *C. thermautotrophica* strains that are similar to *C. hydrogenoformans* [Ni,Fe]-CODH-III, involved in the Wood–Ljungdahl pathway of acetyl-CoA synthesis from C1 units and low-potential reductants (Svetlitchnyi et al., 2004; Wu et al., 2005), are encoded by CTH_1684 in strain 41^T^ and CFE_1666 in strain 019. The genes of these [Ni,Fe]-CODHs occur in gene clusters (Supplementary Table S2) similar to the *acs* gene clusters of *C. hydrogenoformans* (Wu et al., 2005) and *M. thermoacetica* ATCC 39073 (Pierce et al., 2008). The genes of the Wood–Ljungdahl pathway, which evidently provides for the autotrophic capacity of *C. thermautotrophica* strains, are considered in more detail above, in the subsection Inorganic carbon assimilation.

The [Ni,Fe]-CODHs of *C. thermautotrophica* strains CFE_1617 and CTH_1635 are similar to the *C. hydrogenoformans* [Ni,Fe]-CODH-IV (**Figure 7**), thought to be involved in defense against oxidative stress (Wu et al., 2005). However, these *C. thermautotrophica* [Ni,Fe]-CODH genes differ in their genomic context from the *C. hydrogenoformans* [Ni,Fe]-CODH-IV gene: they are in gene clusters that do not include rubrerythrin gene, which in *C. hydrogenoformans* is thought to be responsible for the final step in a chain of reactions performing hydrogen peroxide reduction to water at the expense of electrons derived from CO. Thus, currently, we do not have any hypothesis about the role of the [Ni,Fe]-CODHs CFE_1617 and CTH_1635.

The remaining fifth and sixth [Ni,Fe]-CODHs of *C. thermautotrophica* strains (CFE_0653, CFE_1919, CTH_0658, CTH_1938) are similar to *C. hydrogenoformans*’ [Ni,Fe]-CODH-V, whose function has not been even hypothetically supposed (Wu et al., 2005). CFE_0653 and CTH_0658 seem to be alone in their operons (judging from the 70-100 bp intergenic spaces upstream and downstream), and CFE_1919 and CTH_1938 are alone judging from the directions of transcription of the neighboring genes. Thus, we are unable to make any predictions about the functions of these [Ni,Fe]-CODHs from purely genomic analysis. Moreover, the genomically lone [Ni,Fe]-CODHs CFE_1919 and CTH_1938 have an altered pattern of the ligands of the active site [Ni-4Fe-5S] C-cluster (Dobbek et al., 2001): they lack the Cys295 residue (*C. hydrogenoformans* CooSII numbering), thought to be important for Ni-coordination in the C-cluster (Inoue et al., 2013).

#### Fe(III) Respiration

Screening of both *C. thermautotrophica* genomes revealed 30 genes in each strain possessing various *c*-type multiheme cytochrome domains. Almost all the encoded proteins are 99–100% identical in the two organisms (Supplementary Table S3). Four of the cytochrome genes comprise typical *nrfAH* loci of dissimilatory nitrite reductase complexes and were excluded from further screening of putative Fe(III) reductases. Some of the rest multiheme cytochrome genes could be involved in extracellular electron transfer chain. We found no homologs of putative quinol oxidizing multiheme CymA, regarded to initiate extracellular electron transfer in *S. oneidensis* (Coursolle and Gralnick, 2010). However, both *C. thermautotrophica* strains possess homologs of inner membrane cytochromes ImcH and CbcL from *G. sulfurreducens*, which have been shown to determine high- and low-potential pathways for metal reduction, respectively (Levar et al., 2017). The homologs of ImcH are encoded by CFE_1714 in strain 019 and CTH_1728 in strain 041^T^, located in identical clusters with two other multiheme cytochrome genes. The homologs of CbcL (Zacharoff et al., 2016) are encoded in two Cbc-like gene clusters in each *C. thermautotrophica* genome. Those are CFE_2192-2193 and CFE_2225-2226 in strain 019 and CTH_2221-2222 and CTH_2327-2328 in strain 041^T^. In each of these clusters, one gene encodes a transmembrane *b*-type diheme domain protein, which could serve for quinol oxidation, and the other encodes a protein with a predicted transmembrane helix and a *c*-type multiheme domain facing the cell surface, which could accept electrons from the *b*-type cytochrome and initiate their further transfer to terminal Fe(III) reductases.

As described above, strain 041^T^ does not reduce any forms of Fe(III). Interestingly, among predicted *c*-type multihemes of *C. thermautotrophica* strains, only one 17-heme cytochrome was identified exclusively in the Fe(III)-reducing strain 019 (Supplementary Table S3). The protein is encoded by CFE_2239 downstream of a 15-heme and a hexaheme *c*-type cytochromes CFE_2242-2243 with unknown function and upstream of two cytochrome *c* maturation proteins and an *S*-layer homology domain-containing protein CFE_2230. The cytochrome CFE_2239 is predicted to contain signal peptide in its C-terminal part and no transmembrane helixes, and thus is likely to be a secreted protein. Topology prediction with CW-PRED service (Fimereli et al., 2012) indicates probable anchoring of CFE_2239 in the cell wall. However, the protein shares no homology with any of the outer surface cytochromes or proteins related to porin-cytochrome complexes that are suggested to perform the final step of extracellular electron transfer in *Shewanella* and *Geobacter* species (Coursolle and Gralnick, 2010; Aklujkar et al., 2013; Shi et al., 2016). The cytochrome CFE_2239 consists of two parts which are likely to have different origin and functions. In the cytochrome CFE_2239, putative catalytic N-terminal part (from 501st to 1486th amino acid residue) harbors all the 17 heme-binding motives organized in several conservative multiheme domains. It has distant homologs among many different multihemes, mainly of proteobacterial origin. Phylogeny reconstruction indicates (Supplementary Figure S3) that this putative catalytic domain could have been acquired by horizontal gene transfer from *Geobacter* species. The C-terminal part of the protein CFE_2239 was not reliably predicted to contain homologs of any conservative protein domains, however, the search against SwissProt database revealed a few weak homologs among bacterial adhesin proteins and eukaryotic secreted proteins involved in receptor-ligand interactions and adhesion.

## Discussion

Bacteria of the genus *Carboxydocella* represent a distinct metabolic group of thermophilic hydrogenogenic carboxydotrophs (Sokolova et al., 2009). The members of this group are either obligately dependent on CO or capable of gaining energy using other catabolic processes (fermentation or different types of anaerobic respiration). Three species of the genus *Carboxydocella* have been isolated from various thermal habitats of Kamchatka peninsula (Sokolova et al., 2002; Slepova et al., 2006; Slobodkina et al., 2012). The metabolism of these organisms differs significantly (Supplementary Table S4). Our new isolate 019, obtained from a core sample near Zavarzin thermal pool at Uzon Caldera, shares some features with all validly described *Carboxydocella* species. However, it belongs phylogenetically to the species with the type strain of which it has the lowest number of common metabolic features: in contrast to strain 019, *C. thermautotrophica* 41^T^ can only grow autotrophically and cannot reduce Fe(III). Two metabolic processes are manifestly common to both *C. thermautotrophica* strains – those are inorganic carbon assimilation and hydrogenogenic carboxydotrophy.

### Diversity of CO-Dehydrogenases

The genomes of *C. thermautotrophica* strains 41^T^ and 019 encode six [Ni,Fe]-CODHs each. Until the recent isolation and genomic study of *Calderihabitans maritimus* (six [Ni,Fe]-CODH genes, including one frameshifted gene (Omae et al., 2017), and the publication of the genome of *Clostridium formicaceticum* (six [Ni,Fe]-CODH genes, CP020559.1, Karl et al., 2017), the highest number of [Ni,Fe]-CODHs encoded in available completely sequenced genomes was five. Our analysis performed in April 2018 by tblastn in the NCBI Complete Prokaryote Genome Database (20,008 genomes) revealed 379 genomes encoding at least one [Ni,Fe]-CODH; of them, 208 genomes encoded more than one [Ni,Fe]-CODH. Six genomes encoded five [Ni,Fe]-CODHs each, and the genome of *Clostridium formicaceticum* encoded six [Ni,Fe]-CODHs. Thus, the genomes of *C. thermautotrophica* strains are top-ranking with respect to the number of [Ni,Fe]-CODHs encoded.

The functional roles of multiple [Ni,Fe]-CODHs have been best studied in *Carboxydothermus hydrogenoformans*, and to four of them functions have been ascribed (Svetlitchnyi et al., 2001, 2004; Soboh et al., 2002; Hedderich, 2004; Wu et al., 2005). Based on comparison of amino acid sequences and genomic contexts, we managed to ascribe functions to only two of the six [Ni,Fe]-CODHs in each of the *C. thermautotrophica* strains and to tentatively suppose a possible role for one more [Ni,Fe]-CODH in each strain.

The [Ni,Fe]-CODH encoded immediately adjacent to ECH genes (apparently in a single operon) is evidently responsible in *C. thermautotrophica* strains for hydrogen formation from CO + H_2_O with transmembrane potential generation. [Ni,Fe]-CODH–ECH gene clusters occur in all of the sequenced genomes of hydrogenogenic carboxydotrophs, the only exception known so far being *Carboxydothermus pertinax* Ug1 (Fukuyama et al., 2017). Three types of such gene clusters have been described, composed of homologous genes that, however, differ in their order and phylogeny (genes specific to a particular cluster type are quite few). The gene cluster found in the genomes of *C. thermautotrophica* strains belongs to the long-known *coo*-type gene cluster. The first [Ni,Fe]-CODH–ECH gene cluster to be described was the *coo* gene cluster of *Rhodospirillum rubrum* S1^T^ (Fox et al., 1996; Kerby et al., 1997). Similar (*coo*-type) gene clusters were then described in *C. hydrogenoformans* Z-2901^T^ (Soboh et al., 2002; Wu et al., 2005) (**Figure 8**), *Thermosinus carboxydivorans* Nor1^T^ (Techtmann et al., 2012), *Desulfotomaculum caboxydivorans* CO-1-SRB^T^ (Visser et al., 2014), *Calderihabitans maritimus* KKC1 (Omae et al., 2017), and mentioned to occur in *Carboxydothermus islandicus* SET (Fukuyama et al., 2017). Gene clusters of *coo*-type also occur in the genomes of some other hydrogenogenic carboxydotrophs (‘*Thermincola potens*’ JR (CP002028.1), *Thermincola ferriacetica* Z-0001^T^ (LGTE00000000)), as well as in some bacteria for which the capacity has not been tested. A peculiar variant of the *coo* gene cluster was described in *Rubrivivax gelatinosus* CBS (Wawrousek et al., 2014). Also known are two other types of [Ni,Fe]-CODH–ECH gene clusters that differ from the *coo* cluster in the order of homologous genes and their phylogeny. One was found in *Caldanaerobacter subterraneus* subspecies (Sant’Anna et al., 2015), and the other was revealed in several representatives of the archaeal genus *Thermococcus* (Lim et al., 2010; Kozhevnikova et al., 2016; Oger et al., 2016). Some of the organisms harboring the gene clusters of the latter two types are known to be capable of hydrogenogenic carboxydotrophy, for others the capacity has not been tested.

Thus, the [Ni,Fe]-CODH–ECH gene cluster peculiar to *C. thermautotrophica* strains belongs to the long-known, best studied, and most widely occurring *coo*-type gene clusters. However, in the *C. thermautotrophica* strains this gene cluster has a more complicated pattern; in particular, it includes a second *cooS* gene in an adjacent and apparently co-regulated *cooSC* operon. This co-regulation, performed by the CO- and redox-sensing transcription regulator protein CooA, provides grounds to speculate that this enigmatic [Ni,Fe]-CODH (CFE_0163 in strain 019 or CTH_0166 in strain 41^T^), which does not have close homologs in *C. hydrogenoformans* or in finished genomes represented in the NCBI nr database, has a role to play in CO oxidation with hydrogen production. It was shown by Soboh et al. (2002) that the CO-oxidizing:H_2_-evolving enzyme complex that these authors isolated from *C. hydrogenoformans* contained both CooS1 and CooS2 at a ratio of about 10:1, although the main function of CooS2 has been proposed to be generation of NADPH for anabolic purposes (Svetlitchnyi et al., 2001). The physiological relevance of the dual composition of the [Ni,Fe]-CODH–ECH enzymatic complex has not been discussed; and we can only mention that CooS1 and CooS2 do not differ significantly in their apparent *K*_m_ values (Svetlitchnyi et al., 2001). No close homologs of CooS2 are encoded in the genomes of *C. thermautotrophica* strains, and it is possible that the discussed ‘enigmatic’ [Ni,Fe]-CODH encoded upstream of the [Ni,Fe]-CODH–ECH gene cluster is a minor substitute in the CO-oxidizing:H_2_-evolving enzyme complex of *C. thermautotrophica.*

None of the other *C. thermautotrophica* [Ni,Fe]-CODHs seem to be co-regulated with the CO-oxidizing:H_2_-evolving enzyme complex, since no CooA-binding sites could be found in proximity of other [Ni,Fe]-CODH genes. Moreover, since the CooA transcription regulator is sensitive both to CO and redox conditions, it may be speculated that the remaining four [Ni,Fe]-CODHs are expressed and active both in the absence and in the presence of electron acceptors other than proton [nitrate for both strains, nitrate or Fe(III) for strain 019]. However, only for one of these [Ni,Fe]-CODHs can we predict function from sequence comparisons with well-studied [Ni,Fe]-CODHs and from genomic context. This [Ni,Fe]-CODH (CTH_1684 and CFE_1666) must be involved in the Wood–Ljungdahl pathway. Interestingly, in some hydrogenogenic autotrophic carboxydotrophs the gene encoding this [Ni,Fe]-CODH becomes frameshifted as a result of cultivation at high CO concentrations. The laboratory-acquired origin of the frameshift and its cause are evident for *C. hydrogenoformans* (Svetlitchnyi et al., 2004; Wu et al., 2005) and can be supposed for *Calderihabitans maritimus*. Svetlitchnyi et al. (2004) showed that in a *C. hydrogenoformans* strain variant with non-frameshifted [Ni,Fe]-CODH, acetyl-CoA synthase existed predominantly as monomer at high CO concentrations and used exogenous CO instead of the CO produced from CO_2_ by the [Ni,Fe]-CODH. Such one-step assimilation of CO should be more beneficial. In the *C. thermautotrophica* strains the [Ni,Fe]-CODHs involved in the Wood–Ljungdahl pathway are not impaired despite repeated culture transfers under 100% CO.

In *M. thermoacetica* the Wood–Ljungdahl pathway plays the roles of both carbon assimilation and energy generation, depending on whether acetyl-CoA is further carboxylated or converted to acetate with ATP formation (Pierce et al., 2008). *C. hydrogenoformans* has been reported (Henstra and Stams, 2011) to switch to acetogenesis from CO under conditions where hydrogen production from CO becomes thermodynamically unfavorable (low substrate and high product concentrations). Both *C. thermautotrophica* strains possess genes closely homologous to those thought to be responsible for the acetogenic growth of *M. thermoacetica* (Schuchmann and Müller, 2014; Basen and Müller, 2017). Especially remarkable is that each of their genomes encodes two methylenetetrahydrofolate reductase homologs and that one of these *metVF* gene variants is clustered with *hdrCBA* and *mvhD* genes, indicative of the formation of the HdrCBA+MetVF+ MvhD methylenetetrahydrofolate-reducing electron-bifurcating complex, which is highly efficient in terms of bioenergetics since it produces low-potential reductant (Mock et al., 2014).

The genomes of each of the *C. thermautotrophica* strains encoded three [Ni,Fe]-CODHs for which we could not predict or suppose a definite function. We can only make some very general comments about the remaining [Ni,Fe]-CODHs. In both strains, particular [Ni,Fe]-CODH(s) may be involved in autotrophic carboxylation reactions that follow acetyl-CoA synthesis, supplying reducing equivalents to carboxylating enzymes, as it was supposed for one of the [Ni,Fe]-CODHs of *Calderihabitans maritimus* based on the genomic proximity of its gene with genes of ferredoxin:oxoacid oxidoreductase (Omae et al., 2017). However, in case of the *C. thermautotrophica* strains, there are no such hints from the genomic contexts.

Anyway, it is evident that CO is an important nutrient in the natural habitat of the *C. thermautotrophica* strains, and they largely base on it their survival strategy.

### Physiology of Fe(III) Reduction

The most intriguing physiological feature of *C. thermautotrophica* strain 019 appeared to be dissimilatory reduction of Fe(III) from minerals, which is obviously coupled to hydrogenogenic CO oxidation. Minerals of Fe(III) are the only form of electron acceptors sustaining *C. thermautotrophica* growth at elevated *E*_h_ of -90 mV (**Figure 1**). Hydrogenogenic carboxydotrophy with protons as electron acceptors was exclusively performed by the organism under strongly reduced conditions (-430 mV). Notably, strain 019 appeared to reduce structural Fe(III) from silica mineral glauconite. This process has not been previously reported in Fe(III) reducers. In our recent report, we have demonstrated oxidation of structural Fe(II) but not the reduction of Fe(III) from glauconite by the alkaliphilic dissimilatory iron-reducer *Geoalkalibacter ferrihydriticus* during acetogenic growth (Zavarzina et al., 2016). In contrast to that finding, *C. thermautotrophica* performed the reduction of structural Fe(III) from the same mineral, as clearly indicated by the formation of siderite (**Figure 5**) and the increase of extractable Fe(II) concentration (**Figure 4**) during strain 019 cultivation with glauconite. Achievement of the highest possible Fe(II) content of glauconite during its reduction is clearly marked by decreased line width of Mössbauer spectra (Supplementary Figure S2, Inlay), which is an indicator of the decrease in the ordering of glauconite crystal lattice. Such disordering results from the attack of Fe^3+^ atoms at the mineral surface, e.g., by bacterial redox systems. Further increase in Mössbauer spectral line width and Fe(II) content of the mineral after the start of cell lysis could be explained by the formation of new solid phase siderite (Supplementary Figure S2). No such changes were observed in abiotic controls, indicating biologically induced process of siderite formation, which, however, is not directly controlled by the redox systems of live cells and proceeds after their lysis.

The reduction of mixed valence Fe(III/II) silica minerals and the oxic Fe(III) mineral ferrihydrite have different impacts on the metabolism of strain 019. When Fe(III) was provided as an electron acceptor in the form of ferrihydrite, cultures of strain 019 revealed two clearly distinguishable growth phases. During the first 24-h phase with high initial *E*_h_ and low initial Fe(II) content, maximal growth rate correlated with maximal rates of Fe(III) reduction, lactate conversion to acetate and low rates of carboxydotrophy and hydrogen formation. The second phase started upon accumulation of Fe(II) and concomitant *E*_h_ decrease. This ‘high Fe(II)’ phase was characterized by pronounced slowdown of growth and Fe(II) formation, but at the same time, the rate of hydrogenogenic carboxydotrophy increased to its maximum.

We may assume that during the first stage of growth both CO and lactate could be simultaneously utilized as electron donors for Fe(III) reduction in parallel with hydrogenogenic carboxydotrophy. Accordingly, three reactions are likely to sustain the growth of strain 019 at this stage:

(1) $${\text{C}}_{3}{\text{H}}_{5}{\text{O}}_{3}{}^{-}\;+\;{\text{H}}_{2}\text{O }+\text{ 4F}{\text{e}}^{\text{3+}}\;\to \;{\text{C}}_{2}{\text{H}}_{3}{\text{O}}_{2}{}^{-}\;+\text{ C}{\text{O}}_{2}\;+\text{ 4F}{\text{e}}^{\text{2+}}\;+\;4{\text{H}}^{+},$$

(2) $$\text{CO }+\;{\text{H}}_{2}\text{O }+\;{\text{2Fe}}^{\text{3+}}\;\to \;{\text{CO}}_{2}\;+\;{\text{2Fe}}^{\text{2+}}\;+\;{\text{2H}}^{+}\text{,}$$

(3) $$\text{CO }+\;{\text{H}}_{2}\text{O }\to \;{\text{CO}}_{2}\;+\;{\text{H}}_{2}$$

Data on metabolite concentrations (**Figure 2**) allow us to propose the following brutto-equation for energy metabolism of strain 019 at the first stage of its growth on CO and lactate with ferrihydrite:

(4) $${\text{C}}_{3}{\text{H}}_{5}{\text{O}}_{3}{}^{-}\;+\text{ 30CO }+\;{\text{31H}}_{2}\text{O }+\;{\text{30Fe}}^{\text{3+}}\;\to \;{\text{C}}_{2}{\text{H}}_{3}{\text{O}}_{2}{}^{-}\;+\;{\text{31CO}}_{2}\;+\;{\text{30Fe}}^{\text{2+}}\;+\;{\text{30H}}^{+}\;+\;{\text{17H}}_{2}$$

Given that each lactate molecule donates 4 electrons to Fe^3+^ and each CO molecule donates 2 electrons to either Fe^3+^ or H^+^, reaction (4) implies that the ratio of carbon monoxide spent for iron(III) reduction and for hydrogenogenesis was 13:17, i.e., about 43% of the consumed carbon monoxide was utilized for Fe(III) reduction during the first phase of growth of strain 019 with ferrihydrite and CO. Protons produced in this reaction could be translocated by type I NADH-dehydrogenase or ECH complexes. During further growth of the culture, when Fe(II) accumulates and *E*_h_ decreases, hydrogenogenic carboxydotrophy becomes much more active and seems to play the major role in the energy metabolism of strain 019 (**Figure 2**). Indeed, redox-control of the [Ni,Fe]-CODH–ECH cluster transcription in *C. thermautotrophica* via CooA regulator could restrict employment of this energy generating complex under high *E*_h_ growth conditions. On the other hand, both *C. thermautotrophica* strains possess four more [Ni,Fe]-CODH clusters which do not depend on CooA and thus on low redox potential. At least three of those could be active at elevated *E*_h_ being coupled to Fe(III) reduction in yet unknown way. Formation of magnetite during cell growth coupled to ferrihydrite reduction decreases *E*_h_ down to -360 mV and switches on the [Ni,Fe]-CODH–ECH energy generating complex at the second, ‘high Fe(II),’ growth phase of strain 019. As the [Ni,Fe]-CODH–ECH complex deals with soluble electron donor and acceptor inside the cell and includes a single step proton translocation via ECH, it outcompetes the more complicated Fe(III)-reducing extracellular electron transfer pathway for electrons derived from CO oxidation. Metabolite concentrations monitoring (**Figure 2**) allows us to propose the following brutto-equation for the ‘high Fe(II)’ phase of strain 019 growth with ferrihydrite, lactate and CO:

(5) $${\text{C}}_{3}{\text{H}}_{5}{\text{O}}_{3}{}^{-}\;+\text{ 270CO }+\;{\text{271H}}_{2}\text{O }+\;{\text{10Fe}}^{\text{3+}}\;\to {\text{C}}_{2}{\text{H}}_{3}{\text{O}}_{2}{}^{-}\;+\;{\text{271CO}}_{2}\;+\;{\text{10Fe}}^{\text{2+}}\;+\;{\text{10H}}^{+}\;+\;{\text{267H}}_{2}$$

This reaction, in contrast to reaction (4), implies that the main portion of carbon monoxide consumed during the second growth phase of strain 019 was spent for hydrogenogenesis, and only 1% of it (3 moles of 270) was utilized for ferrihydrite reduction. This switch of the electron acceptor for CO oxidation from Fe(III) to more accessible but less energetically favorable intracellular protons, together with magnetite accumulation, which restricts the access of the cells to Fe(III) atoms on the mineral surface, decreases the growth rate of the organism at high Fe(II) content, in spite of intensified consumption of CO and lactate.

### Key Role of CO in Fe(III) Reduction

The inability of strain 019 to reduce any forms of Fe(III) with lactate in the absence of CO indicates the key metabolic role of carbon monoxide utilization pathways in the organism. In particular, even lactate utilization is likely linked to [Ni,Fe]-CODH activity. Lactate enters the metabolic network of *C. thermautotrophica* via pyruvate-forming lactate dehydrogenase. Pyruvate can be converted to formate and acetyl-CoA by pyruvate-formate lyase (formate *C*-acetyltransferase, CFE_0574-575). Formate in its turn could further enter Wood–Ljungdahl pathway via formate-tetrahydrofolate ligase (CFE_0088) and finally be condensed with a CO molecule by the ACS complex (Supplementary Table S2) to form acetyl-CoA, which is then converted to acetate with substrate-level phosphorylation (Supplementary Table S2). Previously, acetogenesis from formate and CO via the Wood–Ljungdahl pathway has been proposed for the acetogen *Acetobacterium woodii* (Bertsch and Müller, 2015). *C. thermautotrophica* strains possess all the necessary genes for acetogenesis (Supplementary Table S2), including those of the HdrCBA+MetVF+MvhD electron-bifurcating complex, which does not seem to be crucial when the energy requirements are covered by hydrogenogenesis from CO, but is highly efficient in case of acetogenesis. In our experiments, conditions favoring acetogenesis by *C. thermautotrophica* strain 019 seem to have existed in the high *E*_h_ culture medium, when the activity of [Ni,Fe]-CODH–ECH was restricted by redox potential. However, from purely genomic analysis, it is difficult to predict whether or not the acetyl-CoA synthase of *C. thermautotrophica* strains can directly use exogenous CO under certain conditions. One should also remember that Fe(III) is indispensable electron acceptor for *C. thermautotrophica* at elevated *E*_h_, and that metabolic link between acetogenesis and Fe(III) reduction in this organism remains to be understood.

Of note is the fact that during organotrophic growth with the Fe(III)-mica mineral glauconite there was no acetate production, and maximal rates of growth, carboxydotrophy, lactate consumption and Fe(III) reduction were observed simultaneously. The most probable cause is the ferrous iron content of the mixed valence mineral glauconite. This Fe(II) could act as an immobilized reductant rapidly decreasing redox potential of the culture medium in the close vicinity of mineral particles and thus stimulating redox-controlled carboxydotrophic growth of the organism. Such a decrease of the *E*_h_ value in the medium containing both ferric and ferrous iron mineral forms is enhanced by growing cultures, as revealed in our control experiment with a ferrihydrite/magnetite mixture. In contrast to ferrihydrite, glauconite did not stimulate autotrophic growth of strain 019 on CO (**Figure 1**). This fact indicates that glauconite reduction is not as energetically favorable as ferrihydrite reduction, and Fe(II) production from glauconite is rather caused by secondary activity of the extracellular electron transfer chain of strain 019. The absence of acetate production during growth on lactate with glauconite and CO, as well as the cessation of Fe(III) reduction from glauconite upon exhaustion of CO, indicate that carbon monoxide serves as the only electron donor for the reduction of this mica mineral.

### Genomic Determinants of Extracellular Electron Transfer to Fe(III) Minerals

Utilization of Fe(III) minerals as electron acceptors is challenging and requires extracellular electron transfer chain to be established between enzymatic respiratory system of an organism and Fe atoms on the mineral surface or in the silicate lattice. Crystalline Fe-silicate minerals are less energetically favorable electron acceptors than amorphous or poorly crystalline Fe(III) oxyhydroxides. Nonetheless, the reduction of structural Fe(III) within phyllosilicate clay minerals has been documented for mesophilic, thermophilic and hyperthermophilic microorganisms (Kashefi et al., 2008; Pentráková et al., 2013). However, molecular mechanisms of this process remain poorly understood, as well as no information is still available on microbial reduction of Fe(III) within mica minerals, which are widespread in the Earth’s crust, being precursors of clay minerals in weathering processes. Comprehensively studied are the mechanisms of Fe(III) reduction from oxyhydroxides, such as ferrihydrite. Key genes determining the extracellular electron transfer to these minerals have been revealed and their products have been extensively characterized in model mesophilic proteobacteria (Shi et al., 2016). Extensive data on biochemical mechanisms for Fe(III) oxide reduction have also been obtained for two thermophilic Firmicutes (Carlson et al., 2012; Gavrilov et al., 2012) and three hyperthermophilic archaea of the family *Archaeoglobaceae* (Manzella et al., 2015; Mardanov et al., 2015; Smith et al., 2015), although their pathways for extracellular electron transfer remain less studied than in mesophilic models. In all the mentioned organisms three major groups of multiheme *c*-type cytochromes are considered to drive dissimilatory Fe(III) reduction (Shi et al., 2016), those are: cytochromes associated with the cytoplasmic membrane that accept electrons from the quinone pool of the electron transfer chain; intermediate electron-shuttling cytochromes; and a group of cytochromes associated with the outer membrane (or the *S*-layer), which accept electrons from the shuttles and transfer them to an Fe(III) oxide contacting the cell surface (Xiong et al., 2006; Zhang et al., 2008). Terminal step of electron transfer to Fe(III) oxides is regarded to be provided by porin-cytochrome complexes of pcc-type, first described in *Geobacter* species, or Mtr-type, first described in Fe-reducing *Shewanella* spp. Such complexes, albeit not related phylogenetically to each other, have been recently shown to be widespread among various Fe(III)-reducing and Fe(II)-oxidizing prokaryotes (see White et al., 2016 for review), although it is still unclear whether these complexes are universal for all the prokaryotes reducing Fe(III) from insoluble forms. In our work, comparative genome analysis of two strains of a single species, which differ from each other by the ability to reduce Fe(III), revealed the only gene of a cytochrome which exclusively exists in the genome of the iron-reducing strain 019. Thus, this cytochrome is supposed to determine the ability to reduce Fe(III) from both high-potential iron oxides and low-potential Fe-mica minerals. This gene appeared to encode a secreted multiheme *c*-type cytochrome which shares no homology with any of the components of porin-cytochrome complexes, although its heme-containing part does share homology with some cytochromes of *Geobacter* species. The C-terminus of the putative terminal Fe(III) reductase gene of strain 019 is homologous to secreted adhesins but not to porins or any other beta-barrel structures inherent in porin-cytochrome complexes involved in redox cycling of iron. We assume the putative Fe(III) reductase of strain 019 (CFE_2239) to appear by a fusion of genes encoding an adhesion protein and a cytochrome acquired from deltaproteobacteria by horizontal gene transfer. Most probable source organisms of this cytochrome gene belong to *Geobacteraceae* family (Supplementary Figure S3), well known for their Fe(III) reducing activity. This correlates with ecological data indicating proteobacteria to comprise 3–6% of prokaryotic diversity in Zavarzin hot spring (Gumerov et al., 2011; Rozanov et al., 2014), adjoining the natural habitat of strain 019. However, the reduction of structural Fe(III) from mica minerals has not been previously reported either in geobacters or in any other Fe(III) reducers.

Acquiring an Fe(III) reductase that is able to interact with Fe(III)-mica minerals by *C. thermautotrophica* strain 019 significantly impacts the fitness of this species in an unstable hydrothermal environment. Fe(III) is the only electron acceptor that allows the organism to proliferate at elevated redox values and utilize various organic and inorganic carbon sources and electron donors, depending on their availability. The presence of genes which are supposed to determine high- and low-potential pathways for extracellular electron transfer (ImcH-like and CbcL-like cytochromes) in strain 019 makes the organism less dependent on the form of Fe(III) available. So, upon oxygenation of the environment, enhancing the formation of Fe(III) oxides, the organism can utilize these high-potential electron acceptors both for energy conservation and redox control of its ecological microniche via Fe(II) production. A decrease of redox potential would not inhibit iron reduction in the organism, as its catabolism can be switched to another pathway for extracellular electron transfer – to a low-potential form of the acceptor, namely, to the structural Fe(III) of mica minerals, which are abundant in sedimentary environments. Both pathways for the reduction of Fe(III) minerals could be linked to the electron transfer chain in the cell membrane by CbcL-like quinol-oxidizing multiheme cytochrome complexes. Details of these pathways for Fe(III) reduction are to be studied further.

## Conclusion

Our results improve current knowledge on metabolic features of deeply branching Clostridia, and indicate possible ecological importance of *C. thermautotrophica* in its sedimentary thermal habitats. For the first time dissimilatory reduction of structural Fe(III) from ubiquitous silicate mineral glauconite is described.

Genome analysis provided insights in such ecologically relevant properties of *C. thermautotrophica* as hydrogenogenic CO-trophy and Fe(III) reduction. An outstanding number of CO dehydrogenases and a novel type of putative terminal reductase of insoluble Fe(III) compounds have been identified in the organism.

The variety of [Ni,Fe]-CODHs in both *C. thermautotrophica* genomes is supposed to reflect high affinity of the species to this substrate, which is common gaseous component of sedimentary environments of volcanic origin. Fe(III) reducing ability of strain 019 allows it to couple carboxydotrophy with utilization of this high-potential electron acceptor in anoxic sediments. In a more global scale, coupling of Fe(III) reduction with CO oxidation by *C. thermautotrophica* hardwires the electron flow from carbon monoxide to the mineral constituent of the environment that could be further used as an electrical conductor, facilitating direct interspecies electron transfer (“DIET”), or as an electron-storage material, which supports microbial metabolism of other community members (Shi et al., 2016) and could be called a “biogeobattery.”

The difference in the ability of two *C. thermautotrophica* strains to reduce Fe(III) correlates with peculiar ecological factors encouraging strain 019 to evolve or acquire the determinants of extracellular electron transfer to Fe(III) minerals. While strain 41^T^ was isolated from the surface layer of hot spring sediments (Sokolova et al., 2002), strain 019 was obtained from a transition (anaerobic to aerobic) zone of a core rich in Fe-bearing silicates (Rozanov et al., 2014). Further on, a biofilm-like lifestyle in a sedimentary environment with restricted free volume of the mineral phase and tight cell-to-cell contacts favor horizontal gene transfer (Madsen et al., 2012).

Further ecological studies are needed to assess the distribution of *C. thermautotrophica* and prokaryotes with similar phenotypes in various terrestrial hydrothermal vents, widely represented on the Eurasian continent. This would help to estimate the global role of microbial processes coupling the transformation of carbon monoxide and one of the key components of the Earth’s crust, mica minerals.

## Author Contributions

SG, TS, and EB-O convened the research. ST, TS, DZ, AL, and SG designed the research. TS, DZ, and SG performed the cultivation studies. NC and VR performed the Mössbauer studies. ST, AK, and AT performed the genome sequencing and assembling. AL, ST, SG, and IK performed the genome analysis. ST, TS, DZ, NC, EB-O, IK, AL, and SG wrote the manuscript.

## Conflict of Interest Statement

The authors declare that the research was conducted in the absence of any commercial or financial relationships that could be construed as a potential conflict of interest.

## Footnotes

1 http://weizhongli-lab.org/cdhit_suite/cgi-bin/index.cgi

2 https://github.com/jstjohn/SeqPrep

3 http://www.cbs.dtu.dk/services

4 http://www.psort.org/psortb/

5 http://phobius.sbc.su.se/

6 https://www.ebi.ac.uk/Tools/hmmer/search/hmmscan

7 http://enve-omics.ce.gatech.edu/ani/
