# Supplementary material for: Genomic Insights Into Energy Metabolism of Carboxydocella thermautotrophica Coupling Hydrogenogenic CO Oxidation With the Reduction of Fe(III) Minerals
Source: Front Microbiol. 2018 Aug 3;9:1759. doi: 10.3389/fmicb.2018.01759 (PMC6085454; doi:10.3389/fmicb.2018.01759)

**Genomic insights into energy metabolism of *Carboxydocella thermautotrophica* coupling hydrogenogenic CO oxidation with the reduction of Fe(III) minerals**

**Stepan V. Toshchakov[1], Alexander V. Lebedinsky[2*], Tatyana G. Sokolova[2], Daria G. Zavarzina[2], Alexei A. Korzhenkov[1], Alina Teplyuk[1], Natalia I. Chistyakova[3], Vyacheslav S. Rusakov[3], Elizaveta A. Bonch-Osmolovskaya[2], Ilya V. Kublanov[2], Sergey N. Gavrilov[2 *]**

Running title: ***Carboxydocella thermautotrophica* genomes**

**SUPPLEMENTARY MATERIAL**

(Table S4, Figures S1-3)

**Supplementary Table S1.** The distribution of hits to COG functional categories in the genomes of *C. thermautotrophica* strains.
Presented as a separate MS Excel file.

**Supplementary Table S2.** *Carboxydocella thermautotrophica* Wood-Ljungdahl pathway enzymes and, shadowed, enzymes indicative of acetogenesis, particularly, $H_2$-dependent acetogenesis.
Presented as a separate MS Excel file.

**Supplementary Table S3.** *Carboxydocella thermautotrophica* multiheme cytochromes.
Shadowed are predicted NrfAH-type nitrite-reductase complexes, highlighted yellow is the putative terminal Fe(III)-reductase of strain 019, CFE_2239, highlighted green are multiheme cytochromes encoded in the same locus with CFE_2239.
Presented as a separate MS Excel file.

**Supplementary Table S4.** Key metabolic features of validly described *Carboxydocella* species.

| Caracteristics | *C. thermautotrophica* 019 | *C. thermautotrophica* $41^T$ | *C. sporoproducens* | *C. manganica* |
|---|---|---|---|---|
| Genomic DNA G+C content (mol%) | 49.1 | 49.1 | 49.5 | 50.9 |
| Hydrogenogenic carboxydotrophy | + | + | + | - |
| **Electron acceptors** | | | | |
| Mn(IV) | - | - | - | + |
| Fe(III) | + | - | + | + |
| $KNO_3$ | -* | -* | - | - |
| **Electron donors** | | | | |
| yeast extract | + | - | + | + |
| peptone, tryptone | - | - | - | + |
| molecular hydrogen | - | - | - | + |
| formate | - | - | - | + |
| lactate | + | - | + | + |
| acetate | + | - | - | - |
| pyruvate | + | - | + | + |
| glucose | + | - | + | + |
| sucrose | - | - | - | + |

* – nitrate inhibits autotrophic growth but neither inhibits nor stimulates growth with lactate under 100% CO

**Supplementary Figure S1.** Microphotographs of *C. thermautotrophica* strain 019.
A - TEM image of a cell, B –fluorescent image of acridine-stained cell aggregate on magnetite crystals in stationary-phase iron reducing culture, C – phase contrast image of B.

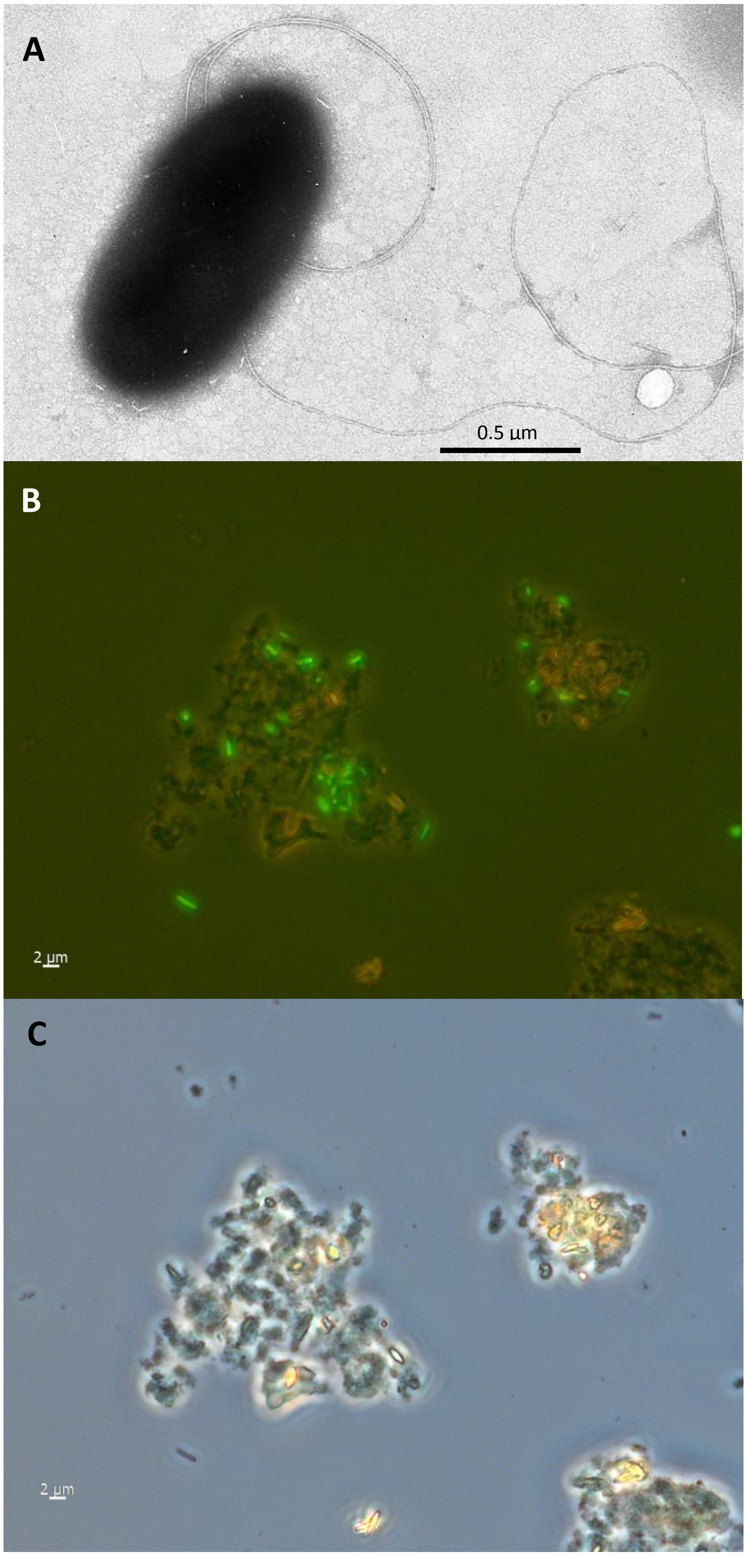

**Supplementary Figure S2.** The relative intensities of subspectra corresponding to different iron atom positions in glauconite in the culture of *C. thermautotrophica* 019 grown with CO and lactate.
Inlay - The spectral line width dependence on cultivation time.

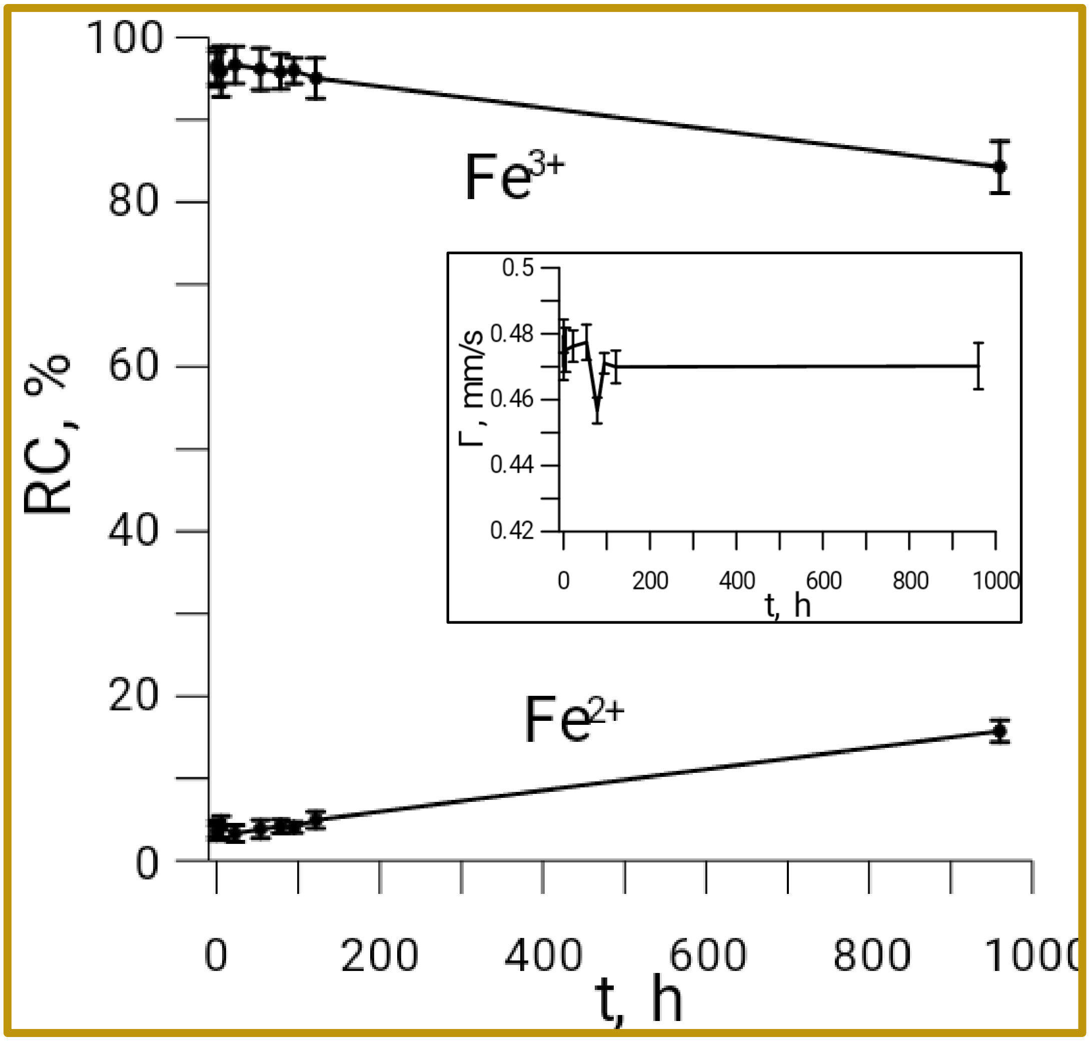

**Supplementary Figure S3.** Condensed phylogenetic tree of the N-terminus of the putative Fe(III) reductase CFE_2239 of *C. thermautotrophica* strain 019.
The tree was constructed by Maximum Likelihood method using MEGA6 (Tamura et al., 2013) at default parameters after aligning sequences with built-in Muscle (default parameters). The original tree was condensed using 40% bootstrap cut-off value in MEGA6.

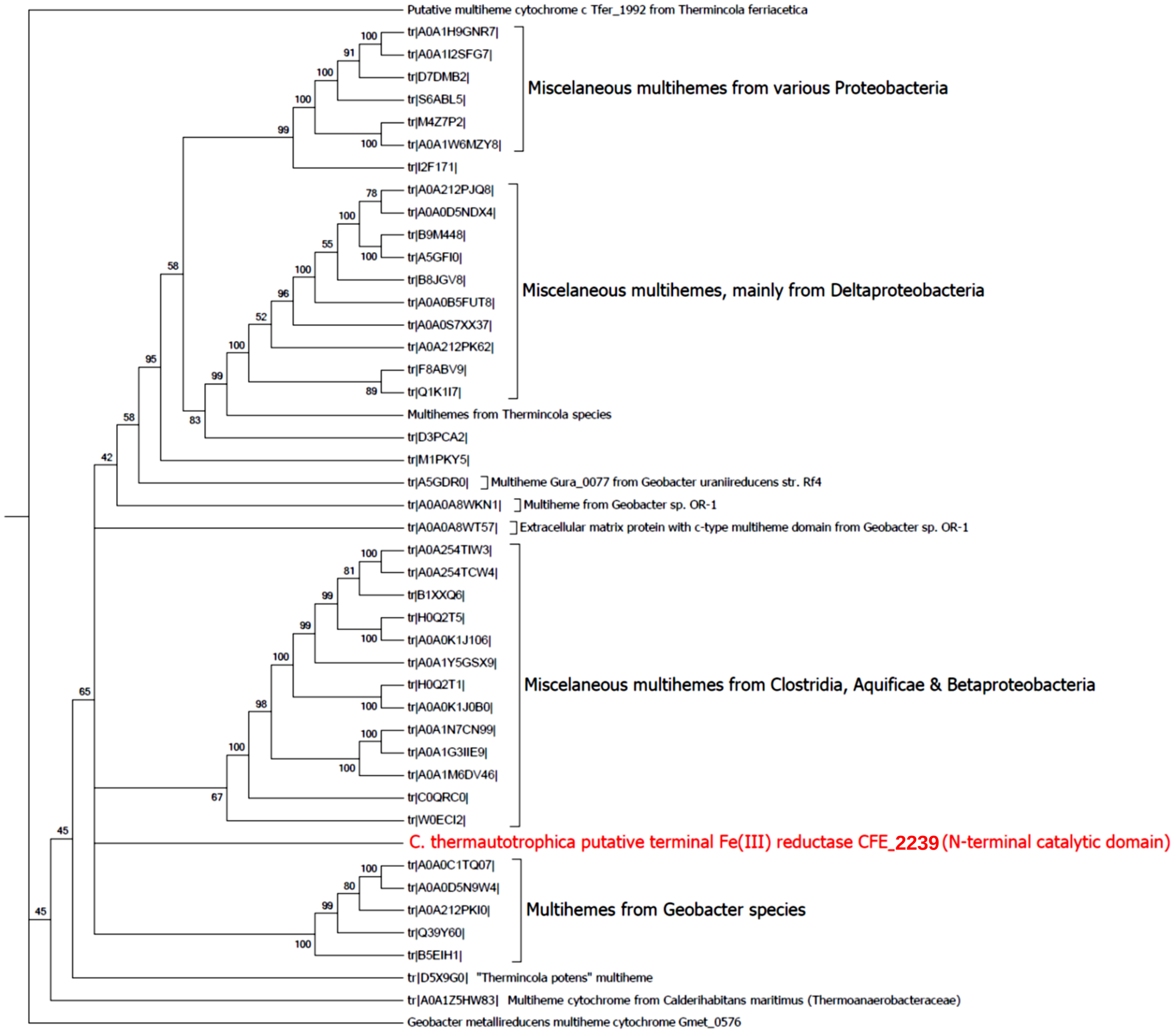

Supplement: Supplementary file 4 [file Data_Sheet_1.PDF]
